# Optimizing polymorphic tomato picking detection: improved YOLOv8n architecture to tackle data under complex environments

**DOI:** 10.3389/fpls.2025.1660480

**Published:** 2026-01-14

**Authors:** Qiang Li, Jie Mao, Pengxin Zhao, Qing Lv, Chao Fu

**Affiliations:** Vocational and Technical College, Hebei Normal University, Shijiazhuang, China

**Keywords:** YOLOv8n, tomato detection, small object detection layer, polymorphic environment, deep learning

## Abstract

**Introduction:**

In modern agriculture, tomatoes, as key economic crops, face challenges during harvesting due to complex growth environments; traditional object detection technologies are limited by performance and struggle to accurately identify and locate ripe and small-target tomatoes under leaf occlusion and uneven illumination.

**Methods:**

To address these issues, this study sets YOLOv8n as the baseline model, focusing on improving it to enhance performance per tomato detection’s core needs. First, it analyzes YOLOv8n’s inherent bottlenecks in feature extraction and small-target recognition, then proposes targeted schemes: specifically, to boost feature extraction, a Space-to-Depth convolution module (SPD) is introduced by restructuring convolutional operations; to improve small-target detection, a dedicated small-target detection layer is added and integrated with the Parallelized Patch-Aware Attention mechanism (PPA); meanwhile, to balance performance and efficiency, a lightweight Slim-Neck structure and a self-developed Detect_CBAM detection head are adopted; finally, the Distance-Intersection over Union loss function (DIoU) optimizes gradient distribution during training. Experiments are conducted on the self-built “tomato_dataset” (7,160 images, divided into 5,008 for training, 720 for validation, 1,432 for testing) with evaluation metrics including bounding box precision, recall, mAP@0.5, mAP@0.5:0.95, Parameters, and FLOPS, and performance comparisons made with mainstream YOLO models (YOLOv5n, YOLOv6n, YOLOv8n), lightweight models (SSD-MobileNetv2, EfficientDet-D0), and two-stage algorithms (Faster R-CNN, Cascade R-CNN).

**Results:**

Results show the improved model achieves 89.6% precision, 87.3% recall, 93.5% mAP@0.5, 58.6% mAP@0.5:0.95, significantly outperforming YOLOv8n and most comparative models, and the two-stage algorithms in both detection accuracy and efficiency.

**Discussion:**

In conclusion, this study solves detection problems of ripe and small-target tomatoes in polymorphic environments, improves the model’s accuracy and robustness, provides reliable technical support for automated harvesting, and contributes to modern agricultural intelligent development.

## 1 Introduction

Tomatoes, a globally cultivated and economically vital crop, are rich in vitamin C, dietary fiber, and other nutrients, offering antioxidant and digestive benefits. They play a crucial role in the fresh consumption and food processing industries (Ali et al., 2020). In 2023, the global tomato market exceeded $50 billion, with an annual production of over 180 million tons. Major producers like China, India, and the United States continue to expand cultivation, driving industry growth. As tomato cultivation continues to expand in scale, the inefficiencies and labor-intensive drawbacks of traditional manual harvesting have become increasingly apparent. Concurrently, intensifying global aging trends have led to worsening labor shortages across agricultural sectors (Yoshida et al., 2022). Furthermore, delayed harvesting of mature fruits results in significant yield and quality reductions due to ethylene diffusion and nutrient competition (Alexander and Grierson, 2002). These compounding factors collectively render traditional manual harvesting inadequate for large-scale production demands. Driven by the rapid advancement of electronics and computer science, machine-vision techniques are progressively supplanting traditional manual inspection in labor-intensive horticultural operations such as fruit and vegetable detection and harvesting (Hou et al., 2023). In the specific context of intelligent tomato picking, the technology leverages non-contact sensing, high-precision feature extraction, and fully automated decision-making pipelines. This paradigm shift not only elevates operational efficiency to an unprecedented level, under its non-destructive and highly robust nature, but also constitutes the core impetus propelling the modernization of precision agriculture.

Thus, developing an efficient, accurate, mature tomato detection framework is key to automated harvesting. It can precisely locate fruits in highly polymorphic greenhouses, cutting identification errors and labor costs. Large-scale deployment of machine-vision-based tomato detection will speed up the agricultural production’s shift to full automation and intelligence, boosting operational precision and throughput while reducing manual dependence. Most importantly, this refined fruit-identification tech optimizes resource use efficiency, benefiting sustainable agriculture and environmental conservation (Botero-Valencia et al., 2025).

Despite significant progress in tomato object detection, existing methods still face technical challenges in real agricultural scenarios—greenhouses have complex lighting, heavy foliage occlusion, and high target-background similarity, while open fields add variable weather interference, both affecting detection stability and leaving room to improve accuracy and real-time performance. To address this, this study proposes a YOLOv8n-based improved algorithm targeting key issues like dense fruits, foliage occlusion, color confusion, and open-field complex weather interference. Its main contributions are as follows:

1. The SPD (Sunkara and Luo, 2022) convolution module is introduced. This module employs shifted downsampling technology to effectively enhance the model’s feature extraction capability for small objects. Meanwhile, a hierarchical structure dedicated to small-object detection is added, and the PPA (Xu et al., 2024) mechanism is integrated to further improve the detection accuracy of small-sized and ripe tomatoes.
2. The lightweight Slim-Neck (Li et al., 2024) structure is integrated, which, by optimizing the feature fusion and information transmission mechanisms, significantly reduces the computational load and parameter scale while maintaining model performance. This design adapts to the requirements of processing complex features in scenarios with foliage occlusion, as it not only reduces the operational burden on embedded terminals but also ensures the effective extraction and transmission of target features in occluded environments, thereby enhancing the detection stability of the model under foliage interference.
3. A self-developed lightweight Detect_CBAM detection head and DIOU loss (Zheng et al., 2019) are incorporated. The Detect_CBAM head optimizes detection accuracy via channel and spatial attention, focusing on fog/snow-obscured tomatoes in open fields. The DIOU loss improves bounding box regression precision and model robustness to polymorphic backgrounds, addressing target-background contrast fluctuations from open-field illumination changes. It compensates for lightweight-related accuracy loss, supports robotic arm grasping, and enhances detection accuracy in illuminated open fields.

## 2 Related work

In recent years, rapid advances in artificial intelligence have revolutionized intelligent agricultural inspection. Within agricultural vision systems, deep-neural-network-based object detection leverages hierarchical feature learning to automatically extract multi-level semantic representations, endowing it with superior performance in crop-fruit detection. Responding to the pressing demands of agricultural modernization, researchers worldwide have converged on deep-learning detection paradigms, focusing on Two-stage and One-stage frameworks. The Two-stage object detection algorithm adopts a phased processing strategy: the first stage generates candidate boxes through a Region Proposal Network (RPN) and filters out potential target regions, while in the second stage, a Convolutional Neural Network (CNN) (Girshick et al., 2014) mines deep features to achieve precise classification and localization of targets.

Scholars have carried out a series of targeted improvements and agricultural application studies based on this framework. For example, Sun et al. (2018) introduced a deep-transfer-learning paradigm for localizing key organs of tomato plants against cluttered backgrounds. Hu et al. (2019) integrated Faster R-CNN (Ren et al., 2017) with an intuitionistic fuzzy set to enable automatic detection of individual mature tomatoes. Widiyanto et al. (2021) leveraged Faster R-CNN for simultaneous maturity classification and detection, demonstrating feasibility within automated ripeness-monitoring pipelines. However, robustness against occlusion, fruit overlap, and variable illumination was not thoroughly validated. Wang et al. (2021) proposed an improved Faster R-CNN tailored for young-fruit detection by incorporating multi-scale feature fusion and an attention mechanism, achieving reliable pre-harvest identification. However, missed detections persist under dense or heavily occluded canopies, and the elevated data and computational demands hinder real-time deployment on resource-constrained devices. Wang et al. (2022) further advanced a refined Faster R-CNN variant-MatDet-for multi-target maturity assessment in complex scenes. The architecture couples a ResNet-50 backbone with a Path Aggregation Network (PANet) (Sandler et al., 2018) and employs RoIAlign to refine bounding-box accuracy. Afonso et al. (2020) applied the Mask R-CNN (He et al., 2017) algorithm to tomato detection in greenhouse environments. Huang et al. (2020) fused fuzzy logic with Mask R-CNN to automatically grade cherry-tomato ripeness levels. Zu et al. (2021) deployed Mask R-CNN with a ResNet50-FPN backbone for simultaneous detection and segmentation of mature green tomatoes in greenhouse environments, specifically addressing color similarity to the background, occlusion, and fruit overlap, yet the intricate model structure incurs slow inference, and small or partially occluded fruits remain prone to omission. Minagawa and Kim (2022) utilized Mask R-CNN to detect tomato clusters and predict individual-fruit harvest timing, integrating the detection output into picking-time decision frameworks.

The One-stage object detection algorithm adopts an end-to-end single-stage processing strategy, eliminating the need to generate candidate boxes. It directly extracts features through the backbone network and simultaneously completes target location prediction and category classification on a single network branch. Thanks to its efficient single-stage architecture, it demonstrates significant advantages in real-time detection scenarios, and in recent years, it has given rise to a series of innovative applications and improved algorithms in the agricultural field. Yuan et al. (2020) introduced the InceptionV2 architecture into the SSD (Liu et al., 2016) framework. By leveraging a parallel extraction mechanism for multi-scale receptive fields, they enhanced the recognition robustness of cherry tomatoes in greenhouse environments. Liu et al. (2022) proposed TomatoDet, an anchor-free detector built upon CenterNet (Law and Deng, 2018), by integrating a convolutional block attention module to enhance feature representation and adopting circular bounding representation to refine tomato-shape fitting. The method markedly improves accuracy and robustness for tomato detection in greenhouse environments. Additionally, the YOLO (You Only Look Once) (Redmon et al., 2016) series of algorithms has garnered widespread attention due to its fast and accurate detection performance, particularly in crop detection, localization, classification, maturity assessment, and automated picking tasks. This advantage is particularly evident in tomato fruit detection and localization tasks. Currently, numerous studies have focused on tomato detection and recognition using models based on the YOLOv8 series, while lightweight solutions optimized based on the YOLOv5 and YOLOv10 series have also emerged, with relevant details shown in **Table 1**.

**Table 1:** Comparison of key features and mAP@0.5 performance of YOLOv8-based models by different authors (N/M-Not mentioned, M-Mentioned).

Reference	Images	Small Target Detection Enhancement Technology	Lightweight Design	Attention Mechanism	Complex Weather Adaptability	mAP@0.5/%
Song et al. (2024)	10180	C2f-Faster bottleneck + MLCA attention	C2f-Faster bottleneck	MLCA attention	N/M	91.3
Fu et al. (2024)	2720	Aux-Head Detection head	EfficientViT Network+C2f-Faster Module	Linear Attention + Cascaded Group Attention	N/M	93.9
Sun (2024)	895	SE Attention Mechanism+α-SimSPPF Module	GSConv_SlimNeck Module	SE Attention Mechanism	N/M	92.5
Huang et al. (2024)	10050	P2 + Hyperparameter optimization	Pruning + Knowledge distillation	N/M	M	84.5
Wang et al. (2024)	6214	C2f-N module + WIoU loss function	Normalization-based Attention Module	Normalization-based Attention Module	N/M	91.4
Liang et al	3000	LAWDarknet53 + SPPFS + Dynamic Head	LAWDarknet53 + SPPFS	Scale-aware + Spatial-aware + Task-aware attention	N/M	95.3
Ours	7160	P2+SPD+PPA	Slim-Neck Module+Detect_CBAM	PPA	M	93.5

Macroscopically, existing models have three key limitations restricting performance breakthroughs. For small target detection enhancement, most methods have clear flaws: while Huang et al. (2024)’s YOLOv8-TD adds a small target detection layer to improve feature extraction, and Solimani et al. (2024) uses SE attention to enhance small target recognition, the former fails to deeply optimize small tomatoes’ spatial correlation and weak features, and the latter focuses on multi-phenotype small target issues without fully exploring cross-scale feature fusion for preserving small target details. Additionally, though Wang et al. (2024)’s NVW-YOLOv8s and Huang et al. (2025)’s AITP-YOLO perform well in multi-ripeness detection or complex scenario adaptation, both lack exclusive enhancement for extremely small tomatoes, leading to poor small tomato detection stability in complex agricultural scenarios.

In terms of lightweight design, existing methods also have common problems: The THYOLO algorithm proposed by Zeng et al. (2023) reconstructs the YOLOv5s backbone network, prunes the Neck layer, and implements NCNN-based quantization. However, its lightweight strategy does not systematically optimize the efficiency of multi-scale feature fusion, and there is still a performance compromise in dense small target detection; the S-YOLO model by Sun (2024) achieves lightweight only through channel pruning and basic module improvement, and fails to deeply solve the problem of feature transmission loss after pruning, resulting in reduced adaptability of the model in greenhouse environments with different planting layouts. Furthermore, the YOLOv8-EA model by Fu et al. (2024) achieves lightweight with the help of the EfficientViT Network and C2f-Faster Module, but faces the problem of increased inference delay due to complex module stacking; the model by Wang et al. (2024) adopts the Normalization-based Attention Module for lightweight, and there is still room for improvement in the coordination between feature optimization and model compression.

In terms of the pertinence of attention mechanism design, most studies only apply attention modules at a “general adaptation” level, lacking customization for tomato detection scenarios. The MLCA attention proposed by Song et al. (2024) does not integrate the spatial distribution features of tomato fruits and branches, leading to insufficient target feature focusing when small tomatoes are densely occluded by leaves. The Linear Attention + Cascaded Group Attention by Fu et al. (2024) fails to adapt to the color and morphological differences of tomatoes at different ripeness stages, easily causing feature confusion between color-turning tomatoes and the background. Sun (2024)’s SE attention only focuses on the channel dimension and ignores dynamic changes in tomato planting density, resulting in inaccurate positioning of small tomatoes in high-density scenarios. In summary, these issues stop attention mechanisms from fully focusing on effective features and suppressing interference, restricting the model’s performance breakthrough in complex agricultural scenarios.

These common issues manifest in more detailed ways in specific studies. Zeng et al. (2023) proposed the lightweight THYOLO algorithm to address redundancy in tomato detection models and heavy hardware reliance. By reconstructing the YOLOv5s backbone, pruning the Neck layer, optimizing hyperparameters, and applying NCNN-based quantization, they developed an Android real-time detection APP. Experiments showed THYOLO outperformed YOLOv5s and other lightweight models, reducing agricultural detection’s dependence on high-performance hardware and supporting low-cost automated picking. However, its dense small-target detection ability and generalization across tomato varieties still need improvement.

Song et al. (2024)’s FastMLCA-YOLOv8 model enhanced the recognition and localization capabilities of string tomato pedicel picking points in complex environments. It achieved this by replacing the bottleneck structure, introducing an attention mechanism, and improving algorithms. Its performance is better than that of YOLOv8. But it faces challenges in handling severely occluded pedicels and acquiring depth information of extremely short pedicels. It also lacks universality for different varieties of string tomatoes.

Fu et al. (2024) proposed the YOLOv8-EA model. It integrates multiple modules to optimize multi-stage tomato detection performance. However, the relatively complex model structure increases training difficulty. In practical applications, it has higher hardware requirements, which are not conducive to deployment on resource-constrained devices.

Sun (2024) developed the S-YOLO model. It significantly improved feature extraction capabilities in complex greenhouse environments. This was done by constructing a lightweight structure, designing improved modules, proposing enhanced algorithms, and integrating attention mechanisms. Yet it has poor adaptability to greenhouse environments with different planting scales and layouts. Its detection performance drops significantly under extreme lighting conditions.

Huang et al. (2024) proposed the YOLOv8-TD model, which enhances feature capture via an added small target detection layer, reduces model scale through pruning, and restores detection accuracy with distillation—achieving a balance of high accuracy, lightweight performance, and real-time capability for tomato detection. However, it exhibits poor accuracy for immature green tomatoes, and its universality remains unvalidated, as its optimization methods have not been applied to other crop detection models to confirm adaptability across agricultural scenarios.

Solimani et al. (2024) addressed imbalanced data and small target recognition in tomato plant multi-phenotype detection. They proposed an integrated solution based on data balancing, with YOLOv8 as the core and SE attention for enhancement. It outperforms Faster R-CNN and YOLOv5x, accurately detecting small targets to support tomato phenotyping and agricultural research. However, it lacks in tomato phenotype semantic differentiation and cross-scenario adaptability.

Wang et al. (2024) proposed a multi-ripeness tomato detection and segmentation solution: it uses a foreground-foreground class balance strategy in data processing, with NVW-YOLOv8s as the core model. It eases semi-ripe sample scarcity, achieving 91.4% detection mAP@0.5, 90.7% segmentation mAP@0.5, and 60.2 fps, providing a reference for tomato yield estimation and multi-ripeness crop detection. However, it lacks a dedicated enhancement mechanism for extremely small tomatoes and has not been validated under extreme lighting or dynamic blur, leading to weak anti-interference capability.

Liang et al. (2025)’s CTDA model enhanced the detection capability of cherry tomatoes in complex environments through specific modules. It outperforms the baseline YOLOv8 model. However, the model’s missed detection rate increases in blurred scenarios. Its anti-interference ability against dynamic image blurring needs urgent improvement. Additionally, the training dataset does not fully cover different growth stages of cherry tomatoes, which may restrict its adaptability in more complex practical scenarios.

Zhao et al. (2025) proposed the Ta-YOLO model based on YOLOv8n. It improved the detection performance of occluded small tomatoes in complex greenhouse environments through multi-module improvements. It achieved an mAP@0.5 of 84.4% on the self-built dataset. However, the model has relatively low detection accuracy for yellow fruits. There are also cases of missed detection for partially occluded fruits in densely planted scenarios. Moreover, the insufficient number of yellow fruit samples in the training data affects its stability in detecting tomatoes of different ripeness levels.

Deng et al. (2025) proposed the YOLOv8-TP model based on YOLOv8n-pose. It realized the synchronous recognition of string tomatoes and their picking points by replacing modules, introducing mechanisms, and enhancing feature extraction capabilities. However, the detection accuracy of this model may decline under extreme lighting conditions. It also faces difficulties in accurately identifying fruits and determining picking points when dealing with highly complex growth postures of string tomatoes.

Huang et al. (2025) proposed AITP-YOLO. It solves small-target missed detection poor complex-scenario generalization and model redundancy in tomato ripeness detection via four-detector head collaboration multi-scale feature fusion Shape-IoU and pruning. It balances accuracy lightweight design and real-time performance providing technical support and a paradigm. However it fails to resolve complex background interference and has weak generalization across tomato varieties/scenarios—its dataset only covers one local Sichuan tomato variety and greenhouse samples excluding open-field other varieties and extreme scenarios.

Extensive research has been done on tomato object detection, but key gaps and unexplored directions remain, directly restricting the practical application of related technologies in agricultural scenarios. First, most current studies rely on a single data source, limiting models’ ability to capture comprehensive features. This is especially problematic for small tomatoes occluded by branches and leaves or color-turning tomatoes similar to the background, where feature extraction lacks integrity and discriminability—polymorphic data fusion could address such information-dimensional limitations. Second, while deep learning ensures high tomato detection accuracy, its high computational cost is notable in mobile and edge environments. Existing lightweight solutions have critical flaws: some sacrifice dense small-target detection for mobile deployment, others require high hardware due to complex module stacking, and still others only use basic pruning without solving feature transmission loss. These issues prevent balancing accuracy, real-time performance, and mobile compatibility, greatly restricting large-scale use in scenarios like field picking robots. Third, most attention mechanisms lack customization for tomato detection and only meet general adaptation needs. Some focus solely on the channel dimension, others are unoptimized for cluster tomatoes’ complex growth, and still others lack exclusive enhancement for extremely small tomatoes. This leads to poor weak-feature capture and interference suppression in complex scenarios, with missed detections of partially occluded fruits in high-density planting. Fourth, existing models have poor scenario adaptability. Most focus on controlled environments like greenhouses and ignore open-field complexities. Under open-field dynamic blur, extreme lighting, heavy rain, or strong light, models either have sharply higher missed detection rates or lower accuracy. When transferred from greenhouses to open fields, they are easily affected by image noise and target feature distortion, significantly reducing robustness.

To address the above issues, this study proposes an innovative tomato detection solution focused on filling research gaps and solving practical pain points, with breakthroughs in three aspects:

First, for insufficient small-target detection accuracy: The P2 layer retains small tomatoes’ basic features to avoid shallow spatial information loss in downsampling; the SPD convolution module extracts weak-feature targets’ fine-grained information via multi-scale spatial decomposition to strengthen cross-layer feature correlations; combined with the PPA attention mechanism, it suppresses background interference, reduces missed detections of extremely small/weak-feature targets, and addresses insufficient attention to small tomatoes’ spatial correlation in traditional methods.

Second, to balance lightweight design and performance: The Slim-Neck strategy streamlines feature fusion paths to cut computational complexity while ensuring multi-scale feature integrity and avoiding post-lightweight feature loss; the self-developed Detect_CBAM detection head optimizes feature screening/weighting from dual attention dimensions, improving target discriminability and compressing parameters to break high hardware requirements. Their combination reduces computational load without accuracy loss, laying a foundation for edge device deployment and balancing lightweight advantages with small-target detection performance.

Third, for insufficient attention mechanism customization and poor scenario adaptability: The PPA attention mechanism focuses on small tomatoes’ weak features—strengthening responses to exclusive features in the channel dimension and locating occluded areas to adapt to complex growth patterns in the spatial dimension. The Detect_CBAM detection head further deepens feature screening by prioritizing high-discriminability channels and enhancing fruit area weights. This dual effect boosts anti-interference in open-field scenarios to solve poor robustness, while compressing parameters, ensuring multi-scale fusion quality, and enhancing feature expression pertinence—avoiding over-generalization and insufficient focus of general attention mechanisms and balancing detection accuracy and inference efficiency.

In summary, the improved YOLOv8n addresses core limitations of existing tomato detection technologies and achieves breakthroughs in small-target accuracy, lightweight-performance balance, scenario-specific attention optimization, and full-scenario robustness. It delivers higher accuracy, stronger environmental robustness, and better generalization in agricultural scenarios, providing an efficient solution for practical agricultural detection and supporting full-scenario intelligent tomato detection. More details on model structure and experimental verification are elaborated in subsequent chapters.

## 3 Data collection and pre-processing methods

### 3.1 Data collection

**Figure 1:**
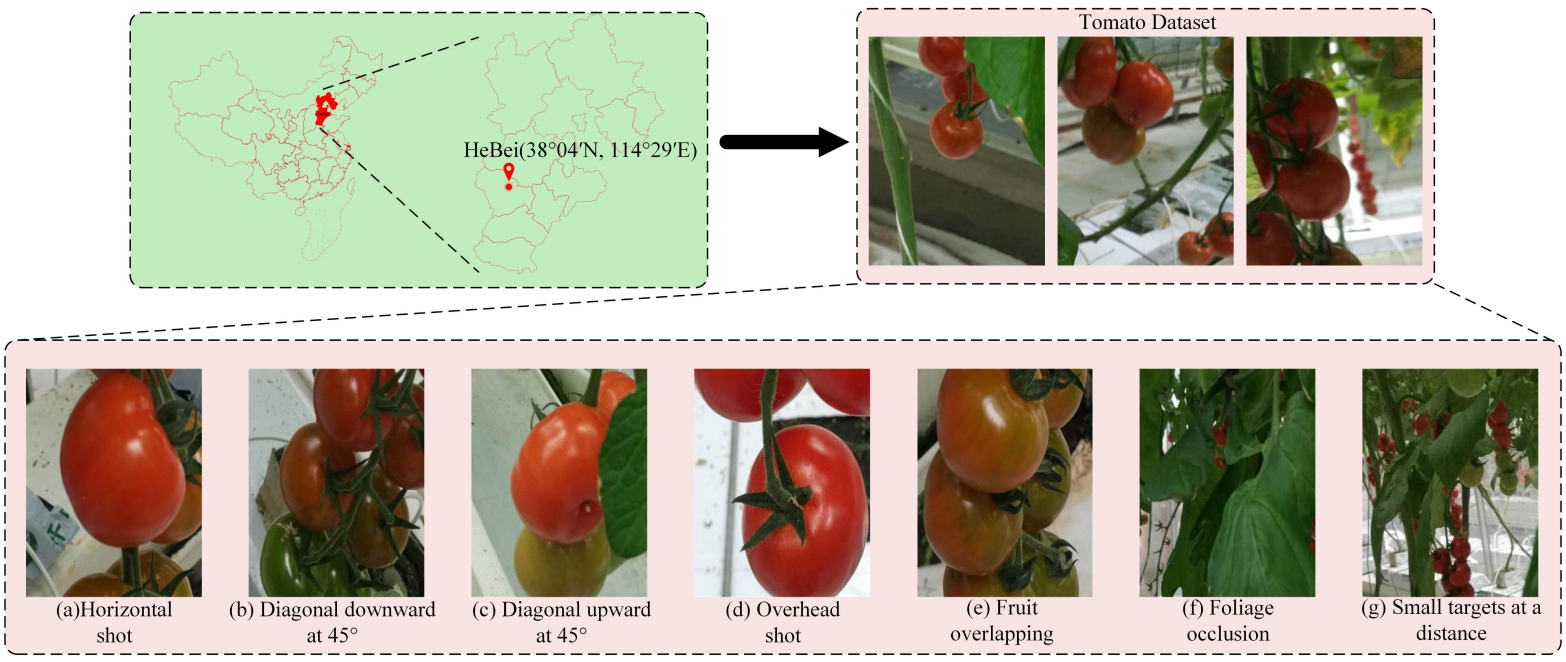
Polymorphic greenhouse tomato dataset, including **(a)** Horizontal shot, **(b)** Diagonal downward at 45°, **(c)** Diagonal upward at 45°, **(d)** Overhead shot, **(e)** Fruit overlapping, **(f)** Foliage occlusion, and **(g)** Small targets at a distance.

This study employs the public tomato dataset ‘Tomato’ from Google Kaggle. This dataset comprises 895 JPG-format images with a resolution of 500×400 pixels that authentically depict greenhouse cultivation environments. The images were captured using 12-megapixel smartphones within glass greenhouses at the National Engineering Research Center for Protected Agriculture, Chongming Base (Li et al., 2020b). They comprehensively represent polymorphic greenhouse environments, including varying shooting angles, occlusions, fruit overlaps, and distant small targets, demonstrating high congruence with real-world agricultural settings. Given its environmental authenticity and scene complexity, this dataset was selected as the initial dataset for model training. The polymorphic greenhouse tomato dataset is shown in **Figure 1**.

### 3.2 Data augmentation and annotation methods

**Figure 2:**
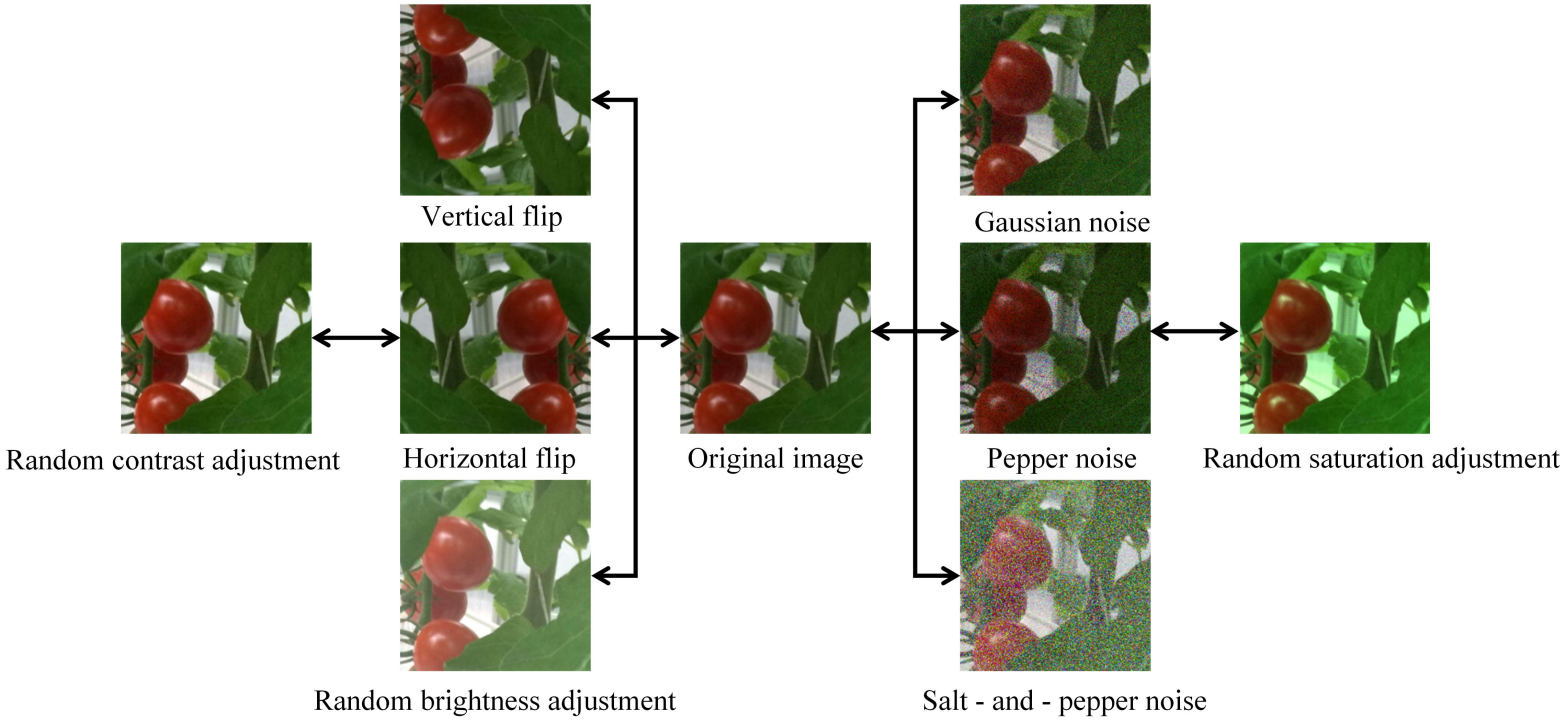
Greenhouse tomato dataset enhancement.

To enhance the recognition accuracy, robustness, and generalization ability of deep learning models for tomato fruits in variable greenhouse environments, and to address issues such as abrupt illumination changes, occlusion, and uneven sample distribution, this study employs data augmentation techniques (Shorten and Khoshgoftaar, 2019) on images to improve the model’s generalization ability and robustness. Vertical flip by 180° and horizontal flip by 180° were used to simulate different observation angles and object inversion scenarios, which effectively enriched sample diversity. Random adjustments of brightness ( ±47%), contrast ( ±50%), and saturation ( ±50%) were made to enhance the distinguishability of image features and improve the model’s adaptability to complex illumination variations. Gaussian noise with a mean of 0 and a standard deviation of 0.1 was added, with the noise amplitude primarily ranging from -0.3 to 0.3, which enhanced the model’s robustness against interference. This helped simulate subtle noise interference in real-world scenarios and strengthen the model’s anti-interference capability. Pepper noise was added, which could set 10% - 30% of pixels to pure black, forming medium-density black dark spots to simulate interference such as local shadows or sensor dead pixels in real scenarios. Salt-and-pepper noise was added, which could set 10% - 30% of pixels to pure white, forming medium-density white bright spots to simulate interference like strong light reflection or camera overexposure. The enhancement effects of some greenhouse tomato images are shown in **Figure 2**.

**Figure 3:**
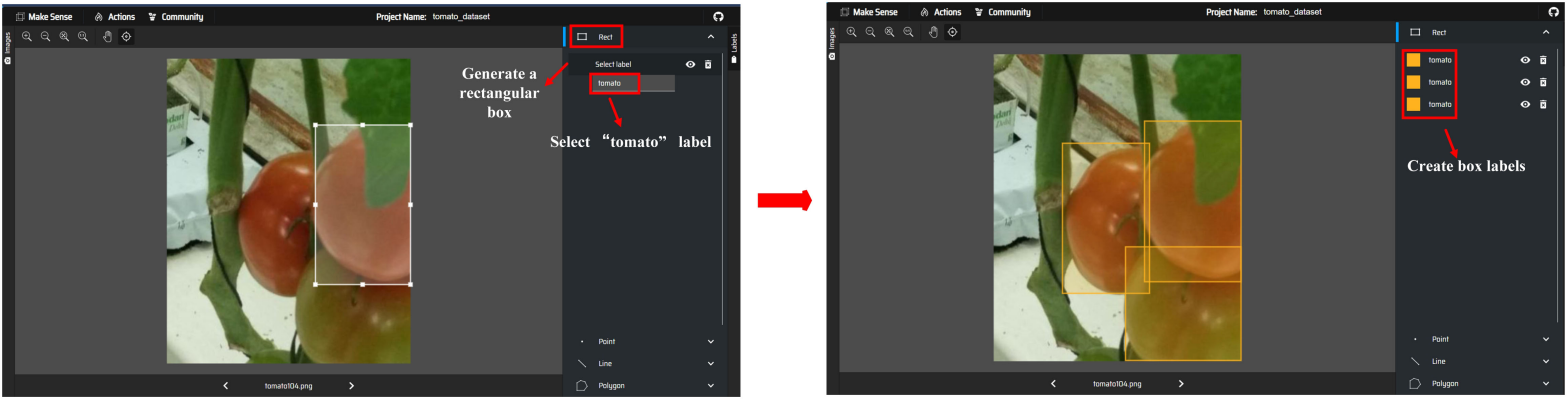
Make Sense’s labeling of tomato targets.

To ensure the reliability and rigor of the dataset, 8 types of data augmentation were applied to each of the training set (626 images), validation set (90 images), and test set (179 images) in the original dataset. Meanwhile, to avoid data bias, the “Make Sense” platform (https://www.makesense.ai/) was used to conduct systematic re-annotation and verification of the original dataset. By clearly defining annotation specifications and unifying annotation standards in advance, the foundation for annotation consistency was established from the data source. On this premise, a mechanism combining annotation by three annotators and random inspection review was adopted. First, three annotators completed the annotation independently. Then, random inspections were conducted to compare the annotation results to correct annotation biases promptly. For ambiguous samples, an additional method of joint discussion by two annotators was adopted to determine the annotation results. Finally, a quality control closed-loop was formed from three dimensions: annotation standards, process control, and special sample handling, which strictly ensured annotation quality. The annotation workflow is shown in **Figure 3**. After the above processing, the “tomato_dataset” containing 7,160 images was finally constructed. It was divided into a training set (5,008 images), a validation set (720 images), and a test set (1,432 images) according to the 7:1:2 ratio of the original dataset.

## 4 Improved YOLOv8n object detection model

### 4.1 YOLOv8 object detection network architecture

YOLOv8 is based on the fully convolutional architecture of YOLOv5 and employs an anchor-free mechanism, consisting of three components: backbone, neck, and head. The backbone and neck introduce the Concatenated 2-layer Fusion (C2f) module to replace the traditional Cross Stage Partial Network with a 3 Convolutional Layers (C3) module, reducing computational load by halving the number of channels. The neck utilizes PANet to fuse multi-scale features, enhancing the integration of high-level semantic features and low-level spatial features. The head adopts a decoupled detection head to separate the regression and prediction branches, minimizing task interference. It also incorporates the Distribution Focal Loss (DFL) (Li et al., 2020a) and Complete-Intersection over Union (CIOU) loss to improve detection accuracy, particularly for bounding box regression in overlapping or low-contrast scenarios.

The YOLOv8 series includes five versions (n/s/m/l/x). Considering the future requirements for real-time performance and deployment convenience, YOLOv8n was chosen in this study as the base model. As the version with the smallest number of parameters and minimal computational load, YOLOv8n is suitable for fast inference in resource-constrained environments, and its concise architecture avoids parameter redundancy in single-class detection tasks. This selection enables efficient real-time detection and easy deployment, aligning with the practical demands of agricultural applications.

### 4.2 YOLOv8n improved model

#### 4.2.1 Space-to-depth convolution module

The performance of current convolutional neural networks (CNNs) has a bottleneck in small object detection. The core reason is that during the traditional downsampling process, strided convolutions and pooling layers directly discard some pixels through the “interval sampling” mechanism, resulting in irreversible loss of fine-grained features. This defect is particularly prominent in greenhouse tomato detection scenarios. Small tomatoes account for an extremely low proportion of pixels in the feature map, and traditional downsampling easily discards key recognition information such as fruit stalks and peel textures, which in turn leads to missed detection. The SPD-Conv module addresses this problem by means of an innovative “spatial-channel dimension rearrangement” mechanism. Its Space-to-Depth (SPD) layer can transfer spatial information to the channel dimension without loss, while the non-strided convolution layer can enhance feature discriminability through learnable parameters. The combination of the two significantly improves the detection accuracy of small tomatoes. In this study, the SPD-Conv module, which consists of an SPD layer and a non-strided convolution layer, is embedded between the Conv module and the C2f module of the backbone network. The specific implementation logic is as follows:

The core of the SPD layer lies in achieving “downsampling without information loss” through “regularized sub-feature map extraction” and “channel dimension concatenation”. First, an intermediate feature map with a size of $S\times S\times C_{1}$ is input, and a series of sub-feature maps are segmented according to the following rules:


$$f_{0,0}=X[0:S:scale,0:S:scale],f_{1,0}=X[1:S:scale,0:S:scale],\ldots ,$$

$$f_{scale-1,0}=X[scale-1:S:scale,0:S:scale];$$

$$f_{0,1}=X[0:S:scale,1:S:scale],f_{1,1},\;\ldots ,$$

$$f_{scale-1,1}=X[scale-1:S:scale,1:S:scale];$$

⋮

$$f_{0,scale-1}=X[0:S:scale,scale-1:S:scale],\;f_{1,scale-1},\;\ldots ,$$

$$f_{scale-1,scale-1}=X[scale-1:S:scale,scale-1:S:scale].$$

**Figure 4:**
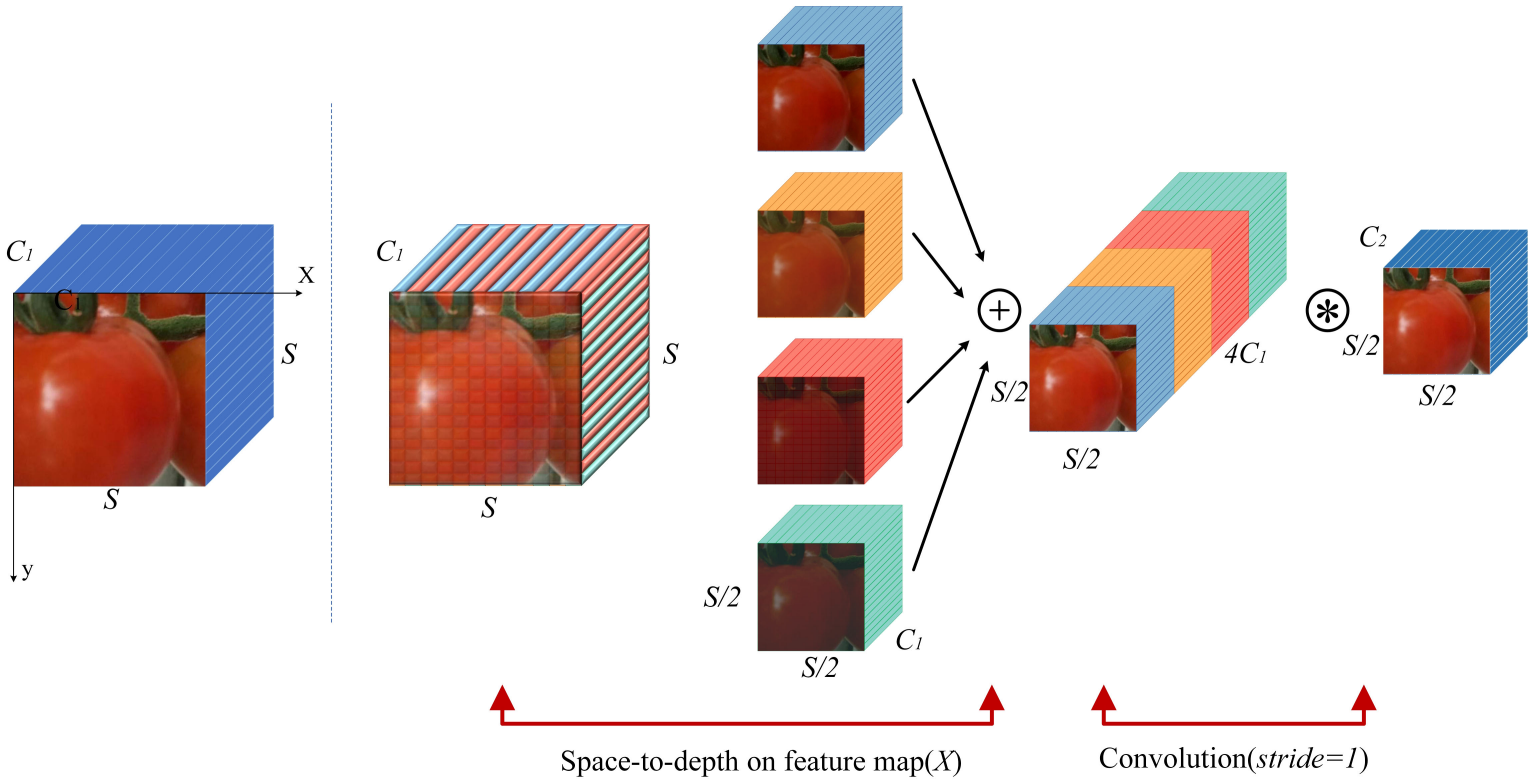
Structure of SPD-Conv with scale = 2.

Moreover, for any original sub-feature map X, the sub-map $f_{\text{x,y}}$ is composed of all elements X(i,j) that satisfy the condition that i+x and j+y are divisible by scale. That is to say, each sub-map downsamples X with scale as the factor. In terms of principle, traditional downsampling directly discards 75% of the pixels, while SPD ensures the integrity of spatial information by means of “extracting all pixels into sub-maps according to divisibility and then concatenating channels”. Every pixel in the input feature map (including the pixels in odd rows and columns that would be lost in traditional downsampling) is assigned to a unique sub-map and is finally preserved through the channel dimension. Specifically, as shown in **Figure 4**, when scale=2 is taken as an example, four sub-maps ${\text{f}}_{0,0}{\text{,f}}_{1,0}{\text{,f}}_{0,1}{\text{,f}}_{1,1}$ are obtained. Each sub-map has a size of $\left(\frac{\text{S}}{2},\frac{\text{S}}{2}{\text{,C}}_{1}\right)$. Then, they are concatenated along the channel dimension to generate a new feature map X'. Its spatial dimension is reduced to $\frac{\text{S}}{\text{scale}}$ with scale as the factor, and the channel dimension is increased to ${\text{scale}}^{2}{\text{C}}_{1}$ with ${\text{scale}}^{2}$ as the factor (i.e., transforming from ${\text{ X(S, S,C}}_{1})$ to $X'\left(\frac{\text{S}}{2},\frac{\text{S}}{2},4{\text{C}}_{1}\right)$. That is, the SPD convolution converts the feature map ${\text{X(S, S,C}}_{1})$ into an intermediate feature map $X'\left(\frac{\text{S}}{\text{scale}},\frac{\text{S}}{\text{scale}}{\text{,scale}}^{2}{\text{C}}_{1}\right)$. After the SPD layer processing, a convolutional layer with a stride of 1 is added. This layer uses ${\text{C}}_{2}$ filters where ${\text{C}}_{2}{\text{<scale}}^{2}{\text{C}}_{1}$, and continues to convert $X'\left(\frac{\text{S}}{\text{scale}},\frac{\text{S}}{\text{scale}}{\text{,scale}}^{2}{\text{C}}_{1}\right)$ into $X''\left(\frac{\text{S}}{\text{scale}},\frac{\text{S}}{\text{scale}}{\text{,C}}_{2}\right)$. In greenhouse environments, tomatoes have the problems of “small size and local occlusion”. The sub-map extraction of SPD can retain the local pixels in occluded areas. For example, for a tomato half-covered by a leaf, the unoccluded half of pixels will be completely extracted into the corresponding sub-map, and subsequent channel concatenation makes this local information aggregate in the channel dimension, facilitating model recognition. Meanwhile, the non-strided convolution layer can compress channels while purposefully preserving discriminative features such as “tomato calyx and red peel”, avoiding the loss of this fine-grained information due to uneven sampling in traditional strided convolution. Thus, it reduces the number of channels while preserving key features. This enhances the model’s ability to extract effective features from small targets, thereby improving the overall performance.

#### 4.2.2 Adding a small target detection layer

In the YOLOv8n model architecture, input images are processed through three feature maps (P3, P4, P5) for contextual association before entering the neck structure, which then outputs target-related information and predicted object locations. These three feature maps have sizes of 80×80 pixels, 40×40 pixels, and 20×20 pixels, respectively, with the core objective of enabling effective detection of targets of different sizes. Specifically, the minimum visible target sizes detectable by the P3, P4, and P5 detection heads are 8×8 pixels, 16×16 pixels, and 32×32 pixels, respectively. However, in practical tomato detection scenarios, due to the small size of some tomato targets, the existing YOLOv8n architecture is prone to missed detections.

To effectively address this issue, this study adds a small-target-specific detection layer P2 to YOLOv8n’s existing three detection layers. The new layer’s 160×160-pixel feature map boosts the network’s focus on small targets and cuts missed detections. This large-scale feature map covers more image context—each element corresponds to a larger original image region, enabling finer target segmentation and better attention to details. By combining shallow positional and deep semantic information, it achieves more accurate small-target localization and identification, offering a more reliable solution for tomato detection.

#### 4.2.3 Parallelized patch-aware attention mechanism

In greenhouse tomato detection, the complex growth environment and varying growth stages cause tomato morphology, color, and other features to be easily disturbed. This leads to partial tomatoes being overlooked or incompletely characterized during conventional feature extraction—single-scale convolution operations either lose local details due to insufficient receptive fields or discard global context due to excessive downsampling. The Parallelized Patch-Aware (PPA) attention mechanism addresses this through a two-tier strategy of multi-branch feature extraction and adaptive attention enhancement: it leverages parallel local, global, and serial convolution branches to comprehensively capture tomato features at different scales, avoiding feature omissions. Meanwhile, its attention mechanism adaptively enhances features critical for tasks like tomato recognition and ripeness judgment, while suppressing interference from backgrounds and irrelevant factors.

The multi-branch feature extraction module is implemented by combining patch-aware operations and multi-level convolution. By adjusting the patch parameter p, the patch-aware process is finely decomposed into three parallel paths: local, global, and serial convolution branches, each focusing on capturing feature information of different scales of the target. Given an input feature tensor $\text{F}\in {\text{ℝ}}^{H'\times W'\times C'}$ (where H', W' are spatial dimensions and C is the channel count), point convolution is first used to preprocess it—this step performs channel dimensionality reduction and reconfiguration without changing spatial resolution, reducing the channel count from C to C'(C'=C/2) while preserving spatial dimensions—obtaining F'∈ℝH'×W'×C'. This not only reduces subsequent computational costs but also compresses redundant channel information, laying a robust and efficient computational foundation for patch splitting. Based on this, the three branches are then executed respectively.

The local and global branches are distinguished by controlling the patch size parameter p, which is achieved by aggregating and rearranging non-overlapping patches in the spatial dimension. Moreover, by calculating the attention matrix between non-overlapping patches, the extraction and interaction of local and global features are enabled. In this process, computationally efficient unfold and reshape operations are adopted to divide F into a set of spatially continuous patches in the form of (p×p,H'/p,W'/p,C). Subsequently, an average operation in the channel dimension is performed to obtain (p×p,H'/p,W'/p), and then a feed-forward network(FFN) (Vaswani et al., 2017) is used for linear calculation: ${\text{P}}_{\text{ffn}}={\text{W}}_{2}\cdot \text{ReLU}\left({\text{W}}_{1}\cdot {\text{P}}_{\text{avg}}+{\text{b}}_{1}\right)+{\text{b}}_{2}$ (where ${\text{W}}_{1},{\text{ W}}_{2}$ are weight matrices, and ${\text{b}}_{1},{\text{ b}}_{2}$ are biases). Finally, an activation function is applied to obtain the probability distribution of linearly calculated features in the spatial dimension, and weights are adjusted accordingly such that these key patches are adaptively selected.

Local branch (p=2): With the help of unfolding and reshaping operations, F' is dissected into a set of spatially continuous patches. For example, if F' has a size of 64×64×C', it is split into 32×32=1024 patches, each covering the core pixel group of a small tomato. Subsequently, a feed-forward neural network (FFN) is used for linear transformation, an activation function derives the probability distribution of linearly calculated features in the spatial dimension, and weights are adjusted accordingly. Finally, ${\text{F}}_{\text{local}}\in {\text{ℝ}}^{H'\times W'\times C'}$ is output, focuses on depicting the local detail features of the target. This design can completely retain visible region pixels of tomatoes occluded by leaves, avoiding missed detections caused by “local pixels being overwhelmed by the background” in traditional convolution.

Global branch (p=4): The operation process is similar to that of the local branch. By processing the patch set, ${\text{F}}_{\text{global}}\in {\text{ℝ}}^{H'\times W'\times C'}$ is output to obtain the global structural features of the target. For densely distributed tomato clusters, the global branch can distinguish “adjacent tomatoes” from “leaf interference” through spatial correlation between patches, reducing false detections and solving the problem of “small tomatoes having a low proportion in feature maps and being easily confused with the background”.

Serial Convolution Branch: Three 3×3 convolutional layers are used to replace the traditional 7×7, 5×5, and 3×3 convolutional layers. The first 3×3 convolution extracts tomato edge features, the second 3×3 convolution fuses edges and local textures, and the third 3×3 convolution captures the overall tomato morphology. These generate three outputs $F_{\text{conv}1}\in {\mathbb{R}}^{\text{H'}\times \text{W'}\times C'}$, $F_{\text{conv}2}\in {\mathbb{R}}^{\text{H'}\times \text{W'}\times C'}$ and $F_{\text{conv}3}\in {\mathbb{R}}^{H'\times W'\times C'}$, which are then summed up to obtain $F_{\text{conv}}\in {\mathbb{R}}^{H'\times W'\times C'}$, realizing the extraction of multi-level features of the target.

The results of the above three branches are aggregated to obtain $\tilde{F}\in {\mathbb{R}}^{H'\times W'\times C'}$. To further enhance the self-adaptability of the features, an attention mechanism is incorporated, which is composed of channel attention and spatial attention in an orderly manner. Specifically, $\tilde{F}$ sequentially undergoes operations with a channel attention map ${\text{M}}_{\text{c}}\in {\text{ℝ}}^{1\times 1\times C'}$ and a spatial attention map ${\text{M}}_{\text{s}}\in {\text{ℝ}}^{H'W'\times 1}$, as shown in Equations 1–3:

(1) $$F_{c}=M_{c}(\tilde{F})⊗\tilde{F}$$

(2) $$\begin{array}{c} F_{s}=M_{s}(F_{c})⊗F_{c} \end{array}$$

(3) $$\begin{array}{c} F''=\delta (B(\text{dropout}(F_{s}))) \end{array}$$

Here, ⊗ represents element-wise multiplication. ${\text{F}}_{\text{c}}\in {\text{ℝ}}^{H\times W\times C'}$ is the feature after channel screening. δ(·) is the linear rectification function (ReLU). B(·) represents the batch normalization (BN) operation, and $F''\in {\mathbb{R}}^{H\times W\times C\prime }$ is the final output of the PPA module.

**Figure 5:**
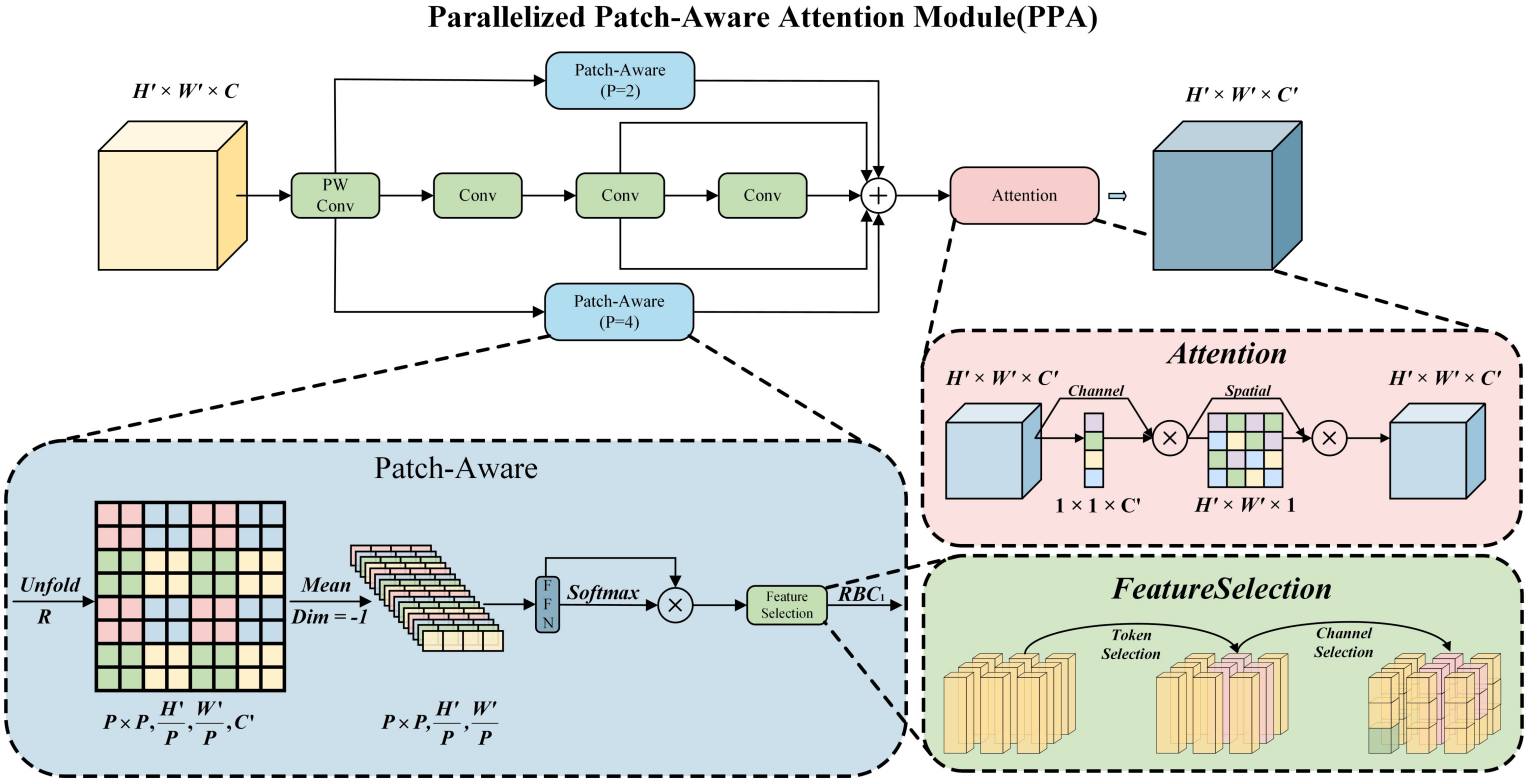
Structure diagram of the PPA attention mechanism.

In the weighting process, let $\text{d}=\frac{H'\times W'}{\text{p}\times \text{p}}$, and the weighting result is expressed as ${\left({\text{t}}_{\text{i}}\right)}_{\text{i}=1}^{\text{C′}}$, where ${\text{t}}_{\text{i}}\in {\text{ℝ}}^{\text{d}}$ represents the i-th output unit. The feature selection process is carried out for each unit, with the output. ${\hat{\text{t}}}_{\text{i}}=\text{P}\cdot \text{sim}\left({\text{t}}_{\text{i}},\text{ξ}\right)\cdot {\text{t}}_{\text{i}}$. Here, $\text{ξ}\in {\text{ℝ}}^{\text{C′}}$, $\text{P}\in {\text{ℝ}}^{\text{C′}\times \text{C′}}$ are specific parameters, and sim(·,·) is a cosine similarity function with values in [0,1]. ξ serves as a task vector to specify task-relevant units. Each unit ${\text{t}}_{\text{i}}$ determines its degree of association with the task vector by measuring the cosine similarity. Channel selection is accomplished via the linear transformation P, followed by reshaping and interpolation to generate the final local feature $F_{local}\in R^{H\times W\times C'}$ and global feature $F_{\text{global}}\in {\mathbb{R}}^{H'\times W'\times C'}$. This mechanism can adaptively adjust feature weights for different tasks — for example, “ripeness judgment” focuses on fruit surface color, and “occluded target detection” focuses on local edges, effectively mitigating performance degradation caused by universal feature extraction. The structure diagram of the PPA attention mechanism is shown in **Figure 5**, where each design component is tailored to address core challenges in greenhouse tomato detection: patch partitioning mitigates “small target detail degradation”, multi-branch fusion enables “scale-adaptive feature capture”, and attention refinement suppresses “background clutter interference”, thereby achieving the objective of “accurate feature extraction with efficient enhancement” in the feature processing pipeline.

#### 4.2.4 Lightweight slim-neck feature fusion network

Adding and improving the model’s small-target detection layer increases computation sharply, reducing overall performance and speed. To cut computing resource use while ensuring accuracy, Slim-Neck’s lightweight GSConv and VoV-GSCSP modules can be used for the lightweight construction of the neck feature fusion network. This eases the cost from increased computation and balances model accuracy, computing resources and operation speed.

##### 4.2.4.1 GSConv module

**Figure 6:**
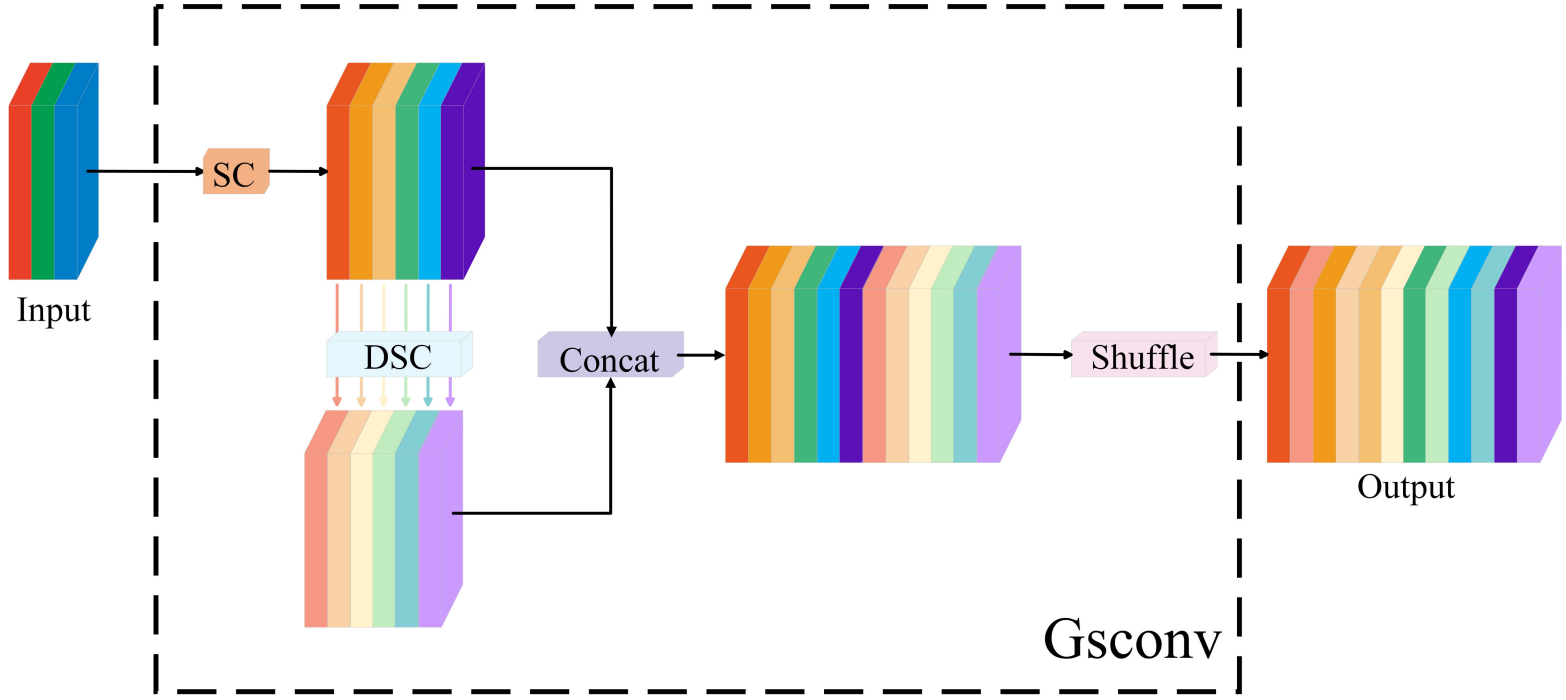
Structure diagram of GSConv convolution.

GSConv comprises three parts: Standard Convolution (SC), Depth-wise Separable Convolution (DSC) and Channel Shuffle. SC extracts rich inter-channel correlated features. DSC captures specific spatial information while cutting computation sharply, and Channel Shuffle fuses features from the two, enhancing feature interactivity and expressiveness. This lets GSConv stay lightweight while having strong feature extraction performance. Its principle (shown in **Figure 6**) is as follows.

The input feature map has *C_1_* channels. First, its channels are split evenly into two parts, each with *C_2_*/2 channels. One part goes to the SC branch, which is a standard 2D convolution layer with batch normalization-2D and activation layers. SC slides a kernel over the feature map to fully capture inter-channel correlated info and retain rich feature details, but has a higher computation cost. The other part enters the DSC branch, which consists of a 2D depth-wise convolution layer with batch normalization-2D and activation layers. DSC first conducts convolution on each channel separately, then fuses channel info via point-wise convolution. This cuts computation greatly, but has insufficient inter-channel info exchange.

The two feature map parts processed by SC and DSC undergo a concat in the channel dimension, restoring the feature map channel count to *C_2_*. Finally, Channel Shuffle is applied to the concatenated feature map to disrupt channel order, allowing features from different branches to permeate and fuse mutually. This enhances feature representation ability while reducing computation and outputs a *C_2_*-channel feature map.

##### 4.2.4.2 VoV-GSCSP module

**Figure 7:**
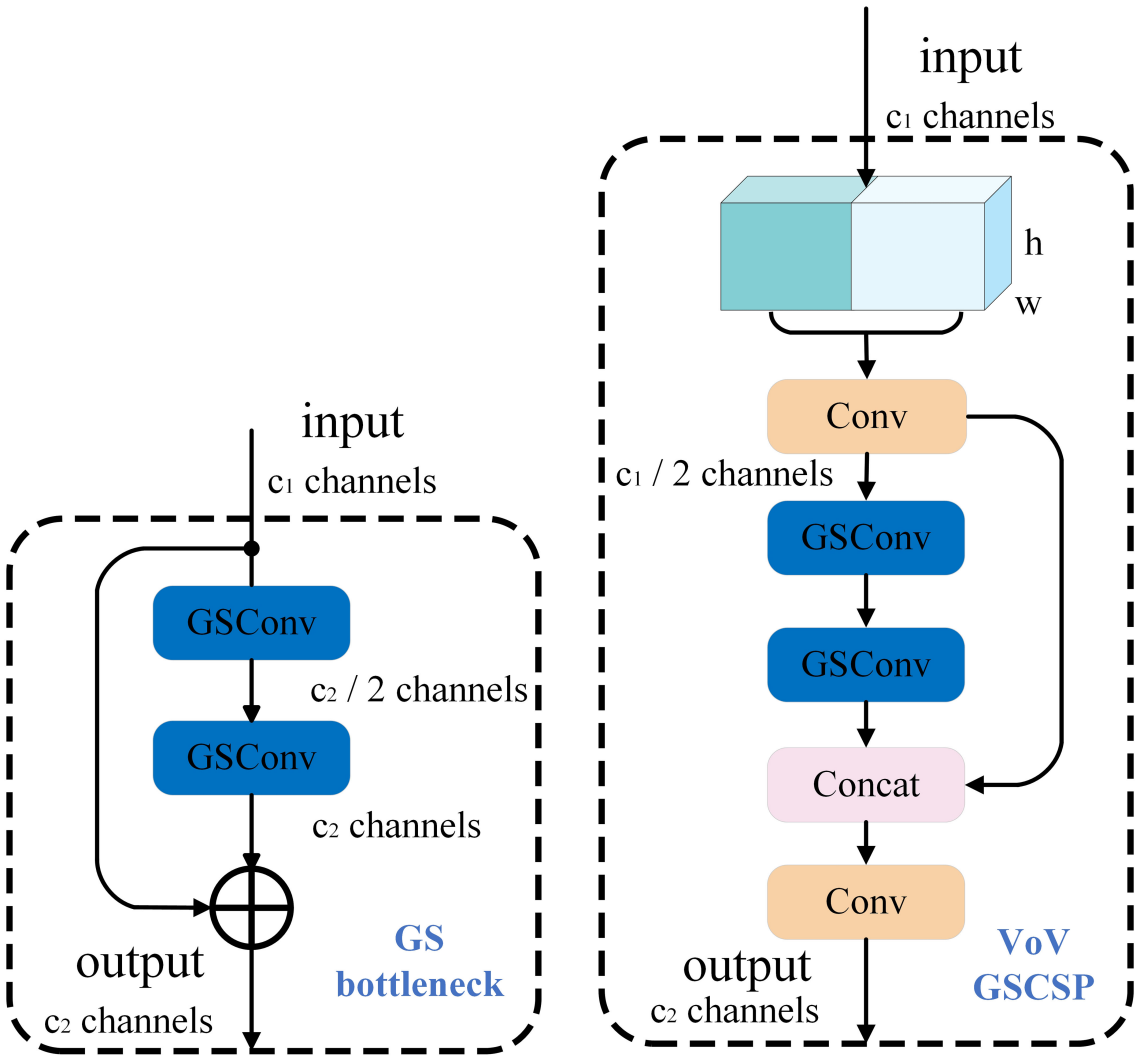
Structure diagram of the VoV-GSCSP module.

To effectively improve model efficiency, this study constructs the GSBottleneck module as shown in **Figure 7** based on GSConv and depth-wise separable convolution. The GSBottleneck consists of two GSConv layers and a standard convolution branch and introduces a residual connection mechanism. After completing the depth-wise separable convolution operation, its output is concatenated and fused with the features of the main branch, which can effectively extract features and control the amount of computation. Subsequently, a cross-stage partial network module, VoV-GSCSP, is designed using a one-time aggregation method, and combined with subsequent convolution processing, it can enhance the model’s perception of multi-channel feature maps. This study uses the VoV-GSCSP module to replace all C2f modules in the neck network. On one hand, it further realizes the lightweight of the model and reduces the computational overhead. On the other hand, it improves the model performance and enhances the feature fusion and detection capabilities.

#### 4.2.5 Improved detection head

To address the accuracy decline caused by lightweight networks and overcome the defects of the existing YOLOv8n detection head, we first analyze its limitations in feature processing. The detection head fails to effectively distinguish the importance of features: feature weights between channels and at spatial positions tend to be uniform. This makes the features of small or occluded tomato targets easily overshadowed by the background or large tomato targets, leaving feature value underutilized and ultimately affecting detection accuracy.

This study proposes the Detect_CBAM detection head. Its core is to integrate the Convolutional Block Attention Module (Woo et al., 2018) into the YOLOv8n detection head structure. With the help of two mechanisms, namely Channel Attention and Spatial Attention, it adaptively enhances features related to the target and suppresses irrelevant background noise. The core idea of this detection head is to use the attention mechanism to dynamically adjust the weights of the feature map in the channel and spatial dimensions, allowing the model to focus on key regions and accurately screen feature information, thereby improving detection accuracy.

**Figure 8:**
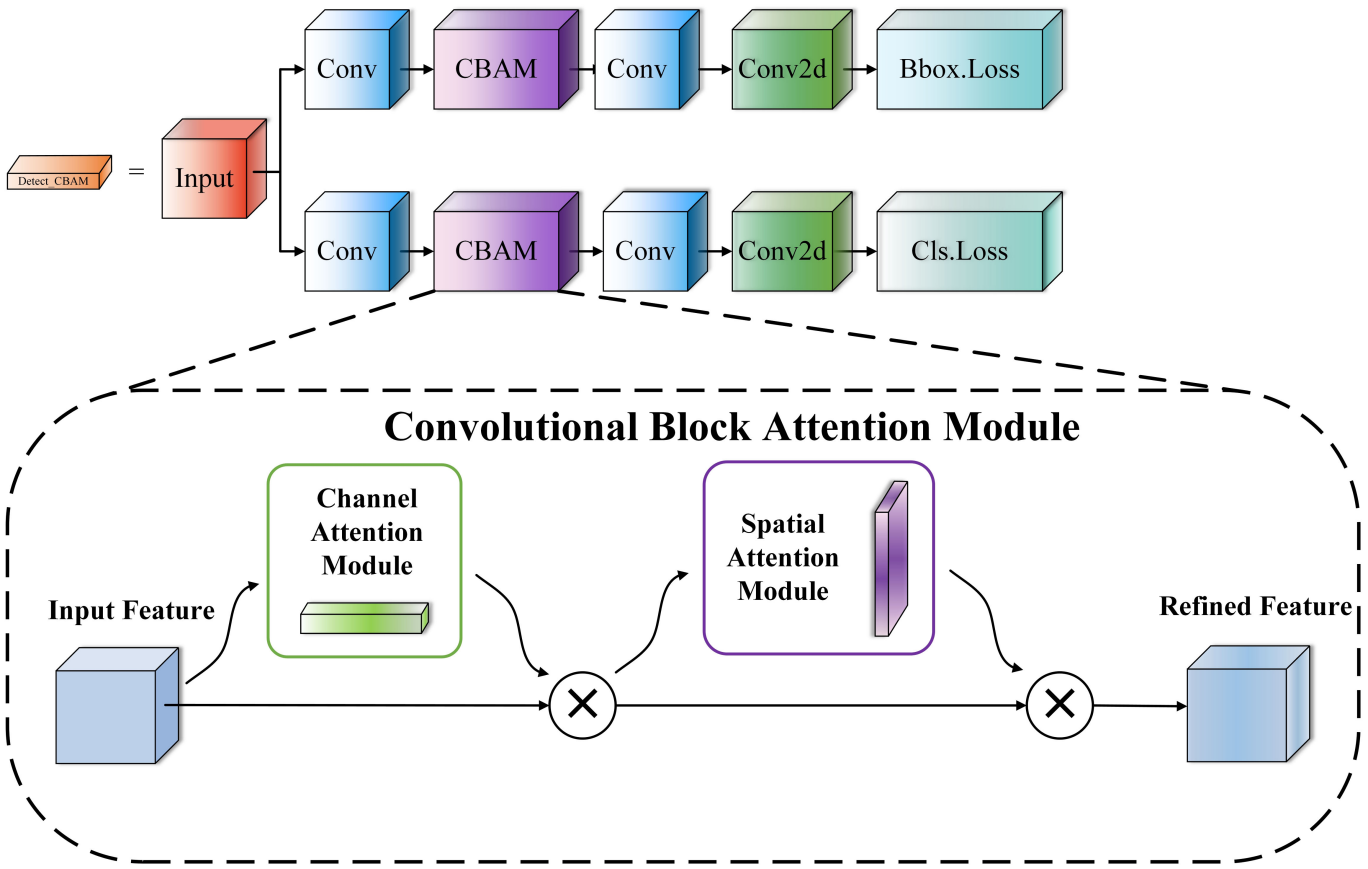
Structure diagram of the Detect_CBAM detection head.

CBAM plays a key role in feature processing: its channel attention module evaluates input feature map channels, aggregates each channel’s feature info via global average/max pooling, and generates weights through a Multi-Layer Perceptron (MLP) to adaptively adjust responses—helping the model focus on target-detection-critical channels. Its spatial attention module creates a spatial descriptor via channel-dimension average/max pooling, gets spatial weights via a convolutional layer to adjust spatial responses, enables accurate target-region focus, effective background suppression, and, for small/occluded targets, precise localization, enhanced feature expression, and prevention of feature overshadowing by background/large targets. This fully uses feature value and boosts detection accuracy. From overall design, the Detect_CBAM head improves performance while keeping the model lightweight: CBAM has a simple structure, few parameters, low computation cost, and no excessive model burden. It improves accuracy by effective feature use without sharp complexity increases, balancing lightweight and high accuracy, with its structure diagram shown in **Figure 8**.

#### 4.2.6 DIoU loss function

The bounding box regression loss function is a critical component of the YOLOv8n model for optimizing the position and shape of bounding boxes, playing a vital role in object detection tasks. It measures the difference between the model’s predicted bounding boxes and the ground-truth bounding boxes—the smaller the regression loss, the more accurately the model can localize object boundaries. As the detection box loss function in YOLOv8, CIoU Loss not only focuses on the overlapping area between the predicted and ground-truth boxes but also considers the consistency of center point distance and aspect ratio. However, it does not achieve the optimal balance between computational complexity and small-target detection performance.

To address this issue, the DIOU loss function is adopted, which abandons the consideration of aspect ratio consistency to reduce computational load. In object detection scenarios, this avoids potential negative effects caused by aspect ratio considerations and enables a more efficient focus on target localization. As a result, the model can capture the positions of tomato targets more quickly and accurately in polymorphic scenarios, effectively improving the recall rate and accuracy of object detection. Additionally, DIoU Loss directly optimizes the normalized distance between the center points of the predicted and target boxes, achieving faster convergence during training and reducing the time and computational resources required for training. The calculation formulas for DIoU Loss are shown in (Equations 4, 5):

(4) $$\begin{array}{c} IoU=\left|\frac{A\cap B}{A\cup B}\right| \end{array}$$

(5) $$\begin{array}{c} Loss_{DIoU}=1-IoU+\frac{\rho^{2}(b,b^{gt})}{c^{2}} \end{array}$$

**Figure 9:**
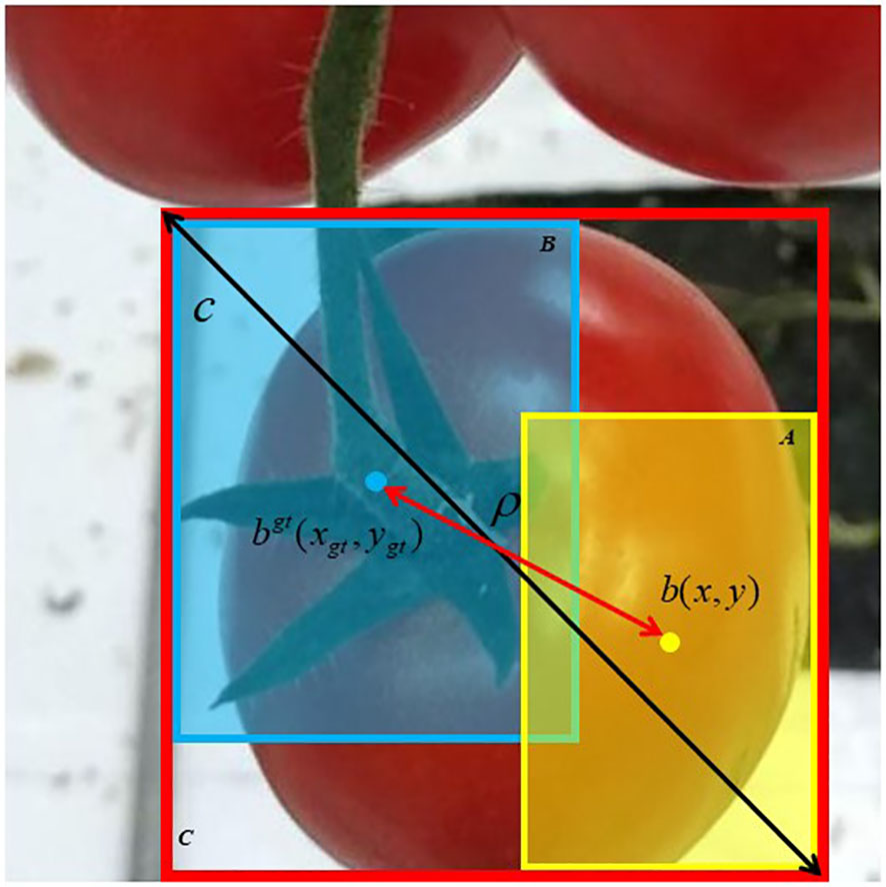
Geometric meanings of DIoU parameters.

Among them, Intersection over Union (IoU) (Yu et al., 2016), namely the intersection-over-union ratio, is a key metric in the field of object detection for evaluating the overlap degree between the predicted bounding box and the ground-truth bounding box. Let A represent the region defined by the predicted bounding box, and B represent the region defined by the ground-truth bounding box. |A∩B| denotes the area of the intersection of the two regions, i.e., the area of the overlapping part between the predicted box and the ground-truth box, |A∪B| represents the area of the union of the two regions, which is the total area after combining the coverage of the predicted bounding box and the ground-truth bounding box. b and ${\text{b}}^{\text{gt}}$ denote the center points of the predicted bounding box A and the ground-truth bounding box B, respectively. ${\text{ρ(b,b}}^{\text{gt}})$ is the Euclidean distance between the center point b of the predicted bounding box and the center point ${\text{b}}^{\text{gt}}$ of the ground-truth bounding box, and c is the diagonal length of the smallest enclosing box C that covers both bounding boxes. The geometric meanings of its parameters are shown in **Figure 9**.

#### 4.2.7 Structure of the improved YOLOv8n network model

The model improvements primarily involve optimizing multiple network components and integrating key mechanisms. In the backbone network, SPD convolution is inserted between the Conv and C2f modules to optimize feature extraction. This enhancement strengthens the capture of detailed features in tomato images, improves the model’s ability to represent polymorphic morphologies and varying lighting conditions, and boosts overall generalization.

In the neck network, C2f modules are replaced with VoV-GSCSP modules, and the PPA attention mechanism is integrated. This combination achieves a lightweight design while mitigating the loss of critical information during downsampling-particularly beneficial for detecting small or occluded tomatoes.

In the head network, a dedicated small target detection layer is added, and the Detect_CBAM detection head is adopted to fuse shallow and deep features. This not only improves tomato detection accuracy and recall but also enhances multi-scale detection capabilities.

**Figure 10:**
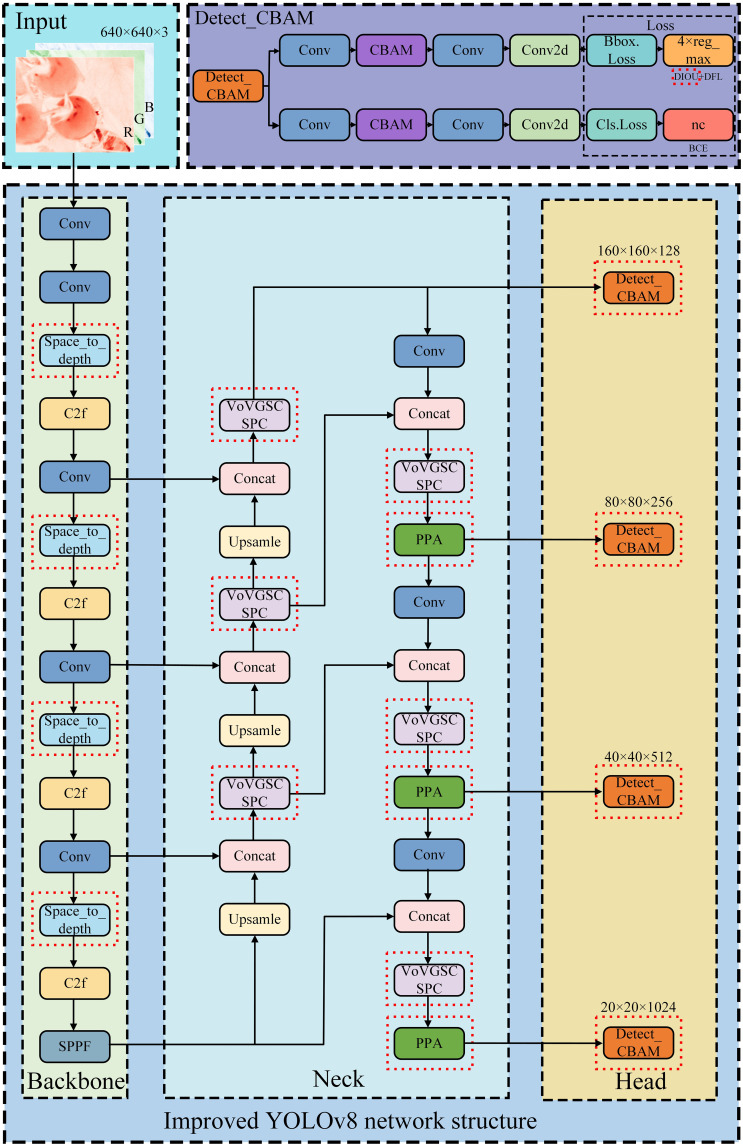
Structure diagram of the improved YOLOv8n network.

Additionally, DIoU Loss is introduced to optimize bounding box regression accuracy, strengthen the model’s robustness under polymorphic and complex backgrounds as well as lighting variations, and ensure high computational efficiency-making it suitable for deployment on resource-constrained devices and facilitating practical detection applications. The architecture of the improved YOLOv8n network is illustrated in **Figure 10**.

## 5 Experiments and result analysis

### 5.1 Experimental environment configuration and parameter configuration

**Table 2** presents the experimental environment configuration for this study, while **Table 3** presents the parameter settings.

**Table 2:** Summary of experimental environment configuration.

Parameter name	Configuration
Operating System	Linux
GPU	NVIDIA GeForce RTX 4090
CPU	Intel (R) Xeon (R) Gold 6152 CPU @ 2.10GHz
Deep-learning Framework	Pytorch1.11.0
CUDA Environment	11.3
Programming Language	Python 3.8
Memory	60GB
Video Memory	24GB

**Table 3:** Summary of key parameter settings for the model’s training process.

Parameter name	Parameter value
epoch	300
batch	32
lr0	0.01
lrf	0.01
momentum	0.937
weight_decay	0.0005
iou	0.7

### 5.2 Experimental evaluation metrics

In this experiment, the evaluation indicators for model performance include bounding box precision (P), recall (R), mAP@0.5, mAP@0.5:0.95, model parameters (Parameters), and floating-point operations (FLOPs). The calculations for precision and recall are shown in (Equations 6, 7):

(6) $$\begin{array}{c} P=\frac{T_{p}}{T_{p}+F_{p}} \end{array}$$

(7) $$\begin{array}{c} R=\frac{T_{p}}{T_{p}+F_{N}} \end{array}$$

${\text{T}}_{\text{p}}$ (true positive) indicates that the target in the image is accurately recognized, and the IoU with the real target exceeds the set threshold. ${\text{F}}_{\text{p}}$ (false positive) indicates that the target is not successfully recognized during the detection process, and the IoU between the detected bounding box and the real target is lower than the set threshold. ${\text{F}}_{\text{N}}$ (false negative) indicates the target that is not detected. The calculation process of the mean Average Precision (mAP) is shown in (Equation 8).

(8) $$\begin{array}{c} mAP=\frac{1}{n}\sum_{i=1}^{N}\int_{0}^{1}P_{n}\left(r\right)\;dr \end{array}$$

Among them, N represents the number of target categories detected in the dataset. mAP@0.5 and mAP@0.5:0.95 are indicators commonly used to evaluate model performance. In this study, unless otherwise specified, mAP defaults to mAP@0.5. The selection of mAP@0.5 (with IoU=0.5) as the core evaluation metric is based on a comprehensive consideration of actual tomato picking scenarios. From the perspective of fruit morphology and robotic arm grasping logic, tomatoes are mostly round or oval in shape, which allows agricultural picking robotic arms to achieve stable grasping as long as they cover 60%-70% of the fruit diameter. IoU=0.5 ensures that the predicted bounding box covers the main area of the fruit, and the flexible structure and force control feedback of agricultural picking robotic arms can compensate for slight positioning deviations. From the perspective of production needs, IoU=0.5 can balance the contradiction between reducing missed detections and avoiding false detections. It can tolerate positioning deviations in scenarios such as leaf occlusion and fruit overlap, thereby controlling the missed detection rate below 8. At the same time, it can filter out invalid predictions that only have edge overlap with the background, keeping the false detection rate within 5%. From the perspective of industry and technical adaptation, IoU=0.5 is a universal threshold for berry crop detection, which has been adopted in studies such as Sun (2024) and Fu et al. (2024). It also aligns with the deployment goal of the lightweight model in this study. Choosing a higher threshold would require a 30% increase in computational complexity, which is inconsistent with the requirement for “embedded device deployment”. mAP@0.5 focuses on the trend of the model’s precision as the recall changes, while mAP@0.5:0.95 comprehensively evaluates the overall performance of the model under various IoU thresholds to accurately reflect the matching degree between the detection boxes and the ground-truth boxes.

### 5.3 Analysis of experimental results

#### 5.3.1 Ablation experiment

To verify the performance enhancement of the improved modules on the model, the same equipment and the “tomato_dataset” dataset are used for training and testing. The experiment designs 12 groups of ablation experiments, as shown in **Tables 4**, **5**. Specifically, **Table 4** presents the experimental data after only adding the small target detection layer, introducing SPD convolution, the VoV-GSCSP module, the PPA attention mechanism, the Detect_CBAM detection head, and adopting DIoU Loss as the loss function. **Table 5** displays the experimental results of the integrated application of the above-mentioned modules. In addition, to further verify the reliability of the results, we repeated the experiment three times on the YOLOv8 model integrated with all improved modules under the same conditions. We calculated the average value ± mstandard deviation of performance metrics such as mAP and precision for each group. This approach helps reduce random errors and improves the stability and credibility of the results. We use the P, R, mAP, number of parameters, and FLOPS of YOLOv8 as the baseline values for the ablation experiments. Among them, “√” indicates that the YOLO model incorporates the module.

**Table 4:** Ablation experiments on YOLOv8n with Individual innovative modules, using precision, recall, mAP, parameters, and FLOPS as baselines.

YOLOv8n	SPD	P2	PPA	VoV-GSCSP	Deteat_CBAM	DIOU	P%	R%	mAP@0.5/%	mAP@0.5:0.95/%	Parameters/ × 10^6^	FLOPS/G
✓	×	×	×	×	×	×	87.6	84.8	90.5	57.3	3.01	8.1
✓	✓	×	×	×	×	×	87.9	84.7	91.5	58.1	3.27	11.5
✓	×	✓	×	×	×	×	90.6	82.7	91.5	55.6	2.92	12.2
✓	×	×	✓	×	×	×	89.6	84.7	91.9	58.4	5.86	12.9
✓	×	×	×	✓	×	×	87.8	84.8	91.7	55.8	2.80	7.2
✓	×	×	×	×	✓	×	87.6	84.6	92.3	56.4	3.01	8.1
✓	×	×	×	×	×	✓	87.8	85.5	92.0	57.4	3.01	8.1

“✓” denotes module incorporation, “×” denotes no incorporation.

**Table 5:** Ablation experiments on YOLOv8n with multiple innovative modules, using precision, recall, mAP, parameters, and FLOPS as baselines.

YOLOv8n	SPD	P2	PPA	VoV-GSCSP	Deteat_CBAM	DIOU	P%	R%	mAP@0.5/%	mAP@0.5:0.95/%	Parameters/ × 10^6^	FLOPS/G
✓	✓	✓	×	×	×	×	91.7	82.7	92.4	59.6	3.20	15.8
✓	✓	✓	✓	×	×	×	88.7	86.7	92.4	59.5	6.00	20.7
✓	✓	✓	✓	✓	×	×	91.2	81.1	91.7	58.6	5.78	19.4
✓	✓	✓	✓	✓	✓	×	90.6	83.6	92.8	58.3	5.78	19.4
✓	✓	✓	✓	✓	✓	✓	89.6 ±0.29	87.3 ±0.15	93.5 ±0.35	58.6 ±0.21	5.78	19.4

“✓” denotes module incorporation, “×” denotes no incorporation.

Based on the ablation experiment data analysis, the improvements to YOLOv8n yield remarkable results in tomato target detection. First, introducing the SPD module significantly increases mAP@0.5 to 91.5% and mAP@0.5:0.95 to 58.1%, enhancing the model’s mean average precision. Adding the small target detection layer boosts bounding box precision by 2.7%, keeps mAP@0.5 at 91.5% (a 1% jump from the baseline), and reduces parameters to 2.92×10^6^. Adding the PPA attention mechanism improves bounding box precision by 2%, mAP@0.5 by 1.4%, and mAP@0.5:0.95 by 1.1%. When only the VoV-GSCSP module is added, the model achieves an mAP@0.5 of 91.7%, with parameters reduced to 2.80×10^6^ and FLOPS decreased to 7.2G. This indicates that the module not only offers lightweight advantages but also effectively optimizes the overall detection precision of the model. While integrating the self-developed Detect_CBAM detection head elevates mAP@0.5 to 92.3%, highlighting its key role in enhancing detection precision. Replacing the original loss function with DIoU Loss increases recall to 85.5% and mAP@0.5 to 92%, demonstrating superior performance in optimizing bounding box regression and overall precision.

When both the small target detection layer and SPD convolution module are incorporated, the model’s bounding box precision rises to 91.7% (a 4.1% improvement over the baseline), and mAP@0.5:0.95 increases by 2.3%. Adding the PPA attention mechanism on this basis further raises recall to 86.7% (a 1.9% gain), significantly improving detection capability. To minimize precision loss and parameter count, adding the VoV-GSCSP module to the pre-improved model reduces recall, mAP@0.5, mAP@0.5:0.95, and floating-point operations but increases bounding box precision by 2.5% compared to the non-lightweight version. To compensate for the resulting decline in some precision metrics, integrating the Detect_CBAM detection head improves recall by 2.5% and mAP@0.5 by 1.1%.

Finally, with the addition of DIoU Loss, the model achieves a bounding box precision of 89.6%, a recall of 87.3%, mAP@0.5 of 93.5%, and mAP@0.5:0.95 of 58.5%, increases of 2%, 2.5%, 3.0%, and 1.2% respectively, over the baseline. Overall, despite increased computation under some configurations, key metrics such as bounding box precision, recall, mAP@0.5, and mAP@0.5:0.95 all show varying degrees of improvement, fully validating the effectiveness of the proposed improvements and significantly enhancing the model’s overall detection performance.

#### 5.3.2 Comparative experiment

##### 5.3.2.1 Comparative experiment of loss functions

In this study, six loss functions were compared with the CIoU loss function used in YOLOv8, evaluating model performance via four metrics: precision, recall, mAP@0.5, and mAP@0.5:0.95. The experimental results are presented in **Table 6**.

**Table 6:** A comparison of six loss functions with YOLOv8’s CIoU is made, assessing performance via precision, recall, mAP@0.5, and mAP@0.5:0.95.

Loss function	P%	R%	mAP@0.5/%	mAP@0.5:0.95/%
CIOU	87.6	84.8	90.5	57.3
DIOU	87.8	85.5	92.0	57.4
EIOU	89.7	82.2	91.4	57.5
GIOU	88.6	84.6	91.6	57.2
SIOU	88.2	82.5	90.6	56.4
inner_CIOU	88.6	81.8	90.5	56.2
inner_SIOU	88.7	82.9	91.0	55.9

As shown in the table, DIoU stands out among loss functions like CIoU and EIoU for its unique advantages. It incorporates the distance between predicted and ground-truth box centers, normalizes this distance using the smallest enclosing rectangle’s diagonal length, and maintains scale invariance. This design gives non-overlapping boxes a clear optimization direction, significantly accelerating model convergence. Experimental data show DIoU achieves 85.5% recall and 92.0% mAP@0.5, confirming its effectiveness in improving target localization accuracy and detection completeness, making it particularly suitable for tomato detection scenarios requiring efficient bounding box regression optimization.

**Figure 11:**
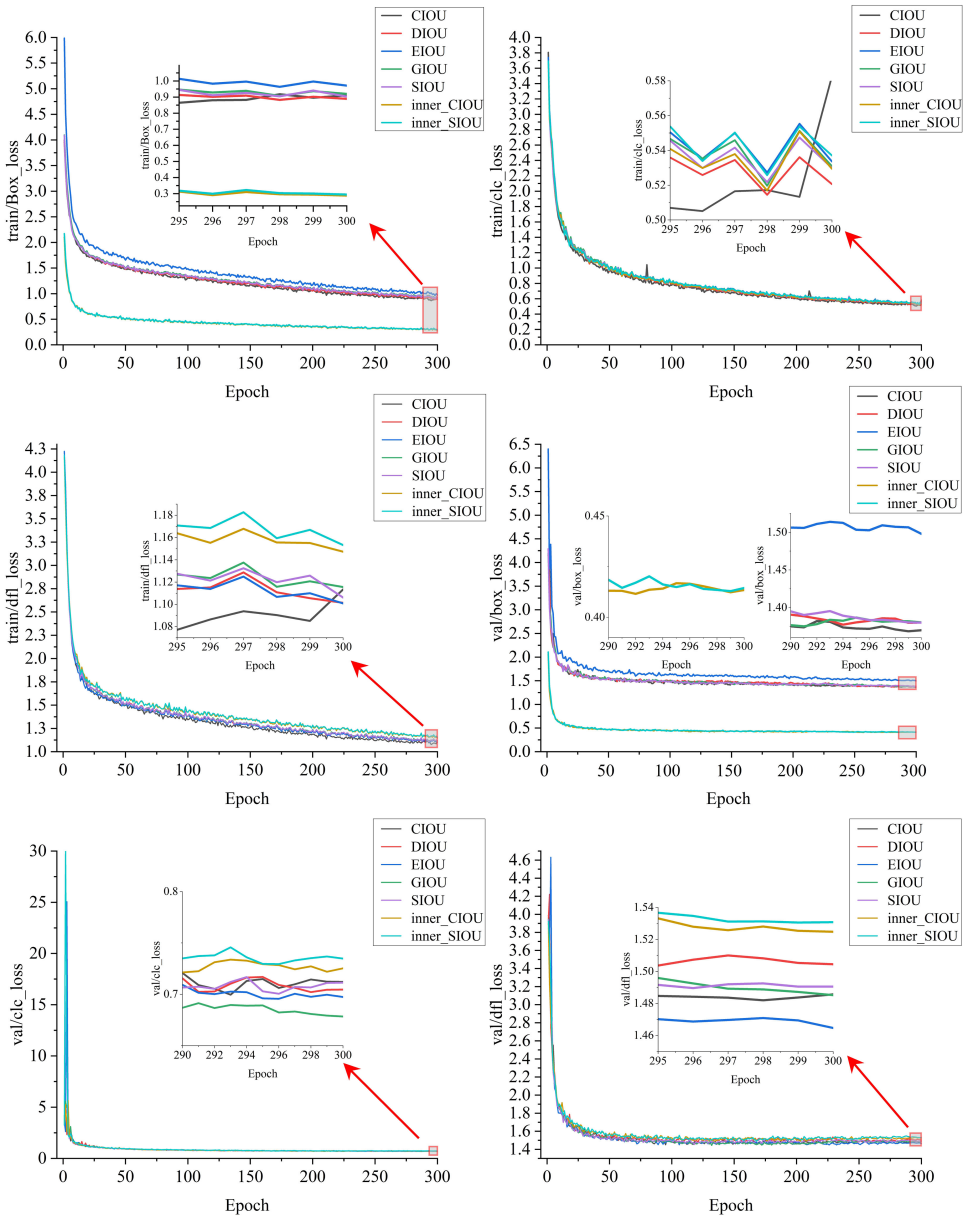
Loss Curves of Various Loss Functions.

Loss value curves were plotted based on the training performance of each loss function, as shown in **Figure 11**. This figure presents curves for seven loss functions (CIoU, DIoU, EIoU, GIoU, SIoU, inner_CIoU, inner_SIoU) across training and validation stages, covering three tasks: bounding box regression (box), classification (cls), and object detection (dfl).

In the training phase’s train/box_loss curve, DIoU exhibits an initial loss value similar to CIoU, with a smooth, orderly decline. While its early-stage decline rate is slightly slower than that of CIoU and EIoU, this stability enables continuous, steady reduction of the gap between predicted and ground-truth bounding boxes during optimization, avoiding training oscillations caused by excessively rapid optimization. In later stages, when other loss functions slow down or fluctuate, DIoU maintains a relatively stable convergence rhythm.

In the train/cls_loss curve, despite an overall tortuous downward trend with multiple fluctuations, DIoU shows smaller fluctuations compared to CIoU and SIoU (which fluctuate significantly). This indicates DIoU can maintain stability in classification loss optimization during training, enabling the model to learn classification features more robustly.

For the train/dfl_loss optimization, DIoU decreases rapidly initially and then slows. The early rapid reduction of object detection-related losses lays a foundation for initial model training, while the downward trend slows in later stages, the convergence effect remains strong, reflecting its sustainability in optimizing object detection losses.

In the validation stage, DIoU’s val/box_loss curve shows a gentle, stable downward trend. Although it lacks the early rapid decline advantage of inner_SIoU, it continuously improves bounding box positioning accuracy on the validation set with a stable optimization rhythm, avoiding performance fluctuations from over-optimization. DIoU retains these stable characteristics in val/cls_loss and val/dfl_loss validation: while not showing absolute dominance at any stage, its consistent performance ensures stability in classification and object detection on the validation set.

In comparison, other loss functions have trade-offs: inner_SIoU, for example, shows obvious advantages in early-stage bounding box loss decline during training and validation, but weakens in later stages. Inner_CIoU maintains good stability in classification loss during training and validation, but lacks outstanding performance in bounding box and object detection loss optimization. In contrast, DIoU’s stability and sustainability across bounding box, classification, and object detection loss optimization-both in training and validation-provide reliable support for model performance improvement at all stages.

##### 5.3.2.2 Lightweight model comparative experiments

**Figure 12:**
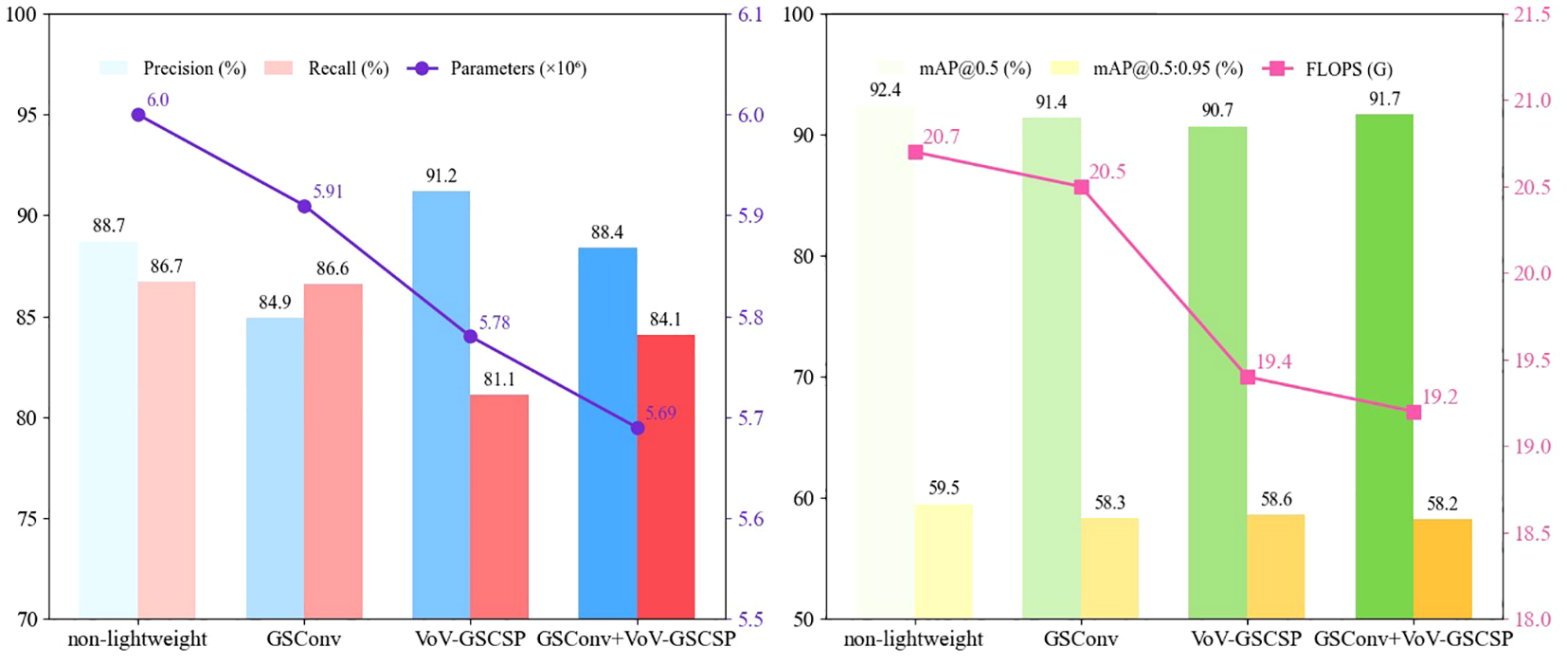
Evaluation metrics of various lightweight models.

In the model architecture, introducing the small object detection layer, SPD convolution module, and PPA attention mechanism adds new network layers, which significantly increases computational complexity. This surge in computation not only slows model inference speed but may also reduce overall detection performance. Therefore, to effectively reduce the model’s demand for computational resources while ensuring the detection accuracy of small objects, the model is made lightweight. The following is a comparative experiment of two modules proposed in the Slim-Neck structure. The experimental results are shown in **Table 7** and **Figure 12**.

**Table 7:** A comparison of lightweight YOLOv8n variant performance with different module combinations—with precision, recall, mAP, parameters, and FLOPS as evaluation indicators.

Lightweight model	P%	R%	mAP@0.5/%	mAP@0.5:0.95/%	Parameters/ × 10^6^	FLOPS/G
YOLOv8n+P2+SPD+PPA	88.7	86.7	92.4	59.5	6.00	20.7
YOLOv8n+P2+SPD+PPA+GSConv	84.9	86.6	91.4	58.3	5.91	20.5
YOLOv8n+P2+SPD+PPA+VoV-GSCSP	91.2	81.1	90.7	58.6	5.78	19.4
YOLOv8n+P2+SPD+PPA+GSConv+VoV-GSCSP	88.7	84.1	91.7	58.2	5.69	19.2

Based on the comprehensive analysis of the above experimental results, in terms of detection accuracy, the model with only the VoV-GSCSP module added has a bounding box precision of 91.2%, mAP@0.5 of 90.7% and mAP@0.5:0.95 of 58.6%, showing good overall detection capability. In terms of parameters and computational load, it has 5.78×10^6^ parameters and 19.4G FLOPS, balancing accuracy and low computational resource needs. Though its 81.1% recall rate is the lowest among the four models, it strikes a better balance between precision and computational consumption—meeting practical detection accuracy requirements without excessive resource usage, making it the optimal choice.

##### 5.3.2.3 Comparative experiments with mainstream models

To comprehensively evaluate the performance of the improved model, this paper conducts comparative experiments with two categories of mainstream models. One category includes current mainstream YOLO series object detection models, such as YOLOv5n, YOLOv8n, YOLOv6n, YOLOv8s, YOLOv8m, YOLOv8l, YOLOv9t, YOLOv10n, and YOLOv11n. The other category covers lightweight object detection models, including SSD-MobileNetv2 (Sandler et al., 2018), SSD-MobileNetv3 (Howard et al., 2019), EfficientDet-D0 (Tan et al., 2020), and Nanodet (RangiLyu, 2021). Additionally, to further verify the advantages of the improved model in small object detection and occlusion scenarios, comparative experiments with two-stage algorithms, namely Faster R-CNN and Cascade R-CNN (Cai and Vasconcelos, 2021), are also carried out. Specific values are shown in **Table 8**.

**Table 8:** A comparison of key metrics for various object detection models in tomato detection tasks, using precision, recall, mAP, parameters, and FLOPS as evaluation indicators.

Mainstream model comparison	P%	R%	mAP@0.5/%	mAP@0.5:0.95/%	Parameters/ × 10^6^	FLOPS/G
Faster R-CNN	38.9	72.8	65.7	26.3	137.10	402.2
Cascade R-CNN	68.6	59.7	89.3	53.0	69.40	196.0
SSD-MobileNetv2	92.4	45.0	73.3	39.3	3.54	6.0
SSD-MobileNetv3	95.4	47.4	80.1	46.4	6.40	1.5
EfficientDet-D0	88.0	66.7	77.9	43.1	3.87	5.2
Nanodet	85.4	55.5	82.4	46.5	1.17	0.5
YOLOv5n	89.6	80.0	90.1	55.4	2.50	7.1
YOLOv6n	90.5	79.3	89.0	55.5	4.23	11.8
YOLOv8n	87.6	84.8	90.5	57.3	3.01	8.1
YOLOv8s	89.1	88.4	92.7	61.6	11.13	28.4
YOLOv8m	90.9	85.5	92.2	63.8	25.84	78.7
YOLOv8l	90.0	84.9	92.2	66.0	39.43	145.2
YOLOv9t	87.0	83.0	90.1	54.8	1.73	6.4
YOLOv10n	87.1	80.4	88.5	54.3	2.69	8.2
YOLOv11n	86.6	84.5	90.2	55.1	2.58	6.3
Ours	89.6 ±0.29	87.3 ±0.15	93.5 ±0.35	58.6 ±0.21	5.78	19.4

In terms of bounding box precision, the improved model reaches 89.6%. This value is higher than that of most mainstream models except SSD-MobileNetv2 and SSD-MobileNetv3. Examples include YOLOv8n (87.6%) and YOLOv8s (89.1%). It is only slightly lower than YOLOv6n (90.5%) and YOLOv8m (90.9%). Faster R-CNN achieves a bounding box precision of 38.9%, and Cascade R-CNN attains a bounding box precision of 68.6%, both lower than that of the improved model, reflecting the effectiveness of the improvements made in our model. This shows that in tomato detection, the model can accurately identify targets with relatively few false detections.

The recall rate reflects the model’s ability to detect all targets. The recall rate of the improved model reaches 87.3%. It significantly outperforms models like SSD-MobileNetv2 (45.0%), SSD-MobileNetv3 (47.4%), EfficientDet-D0 (66.7%), as well as YOLOv5n and YOLOv6n. Faster R-CNN has a recall rate of 72.8%, and Cascade R-CNN has a recall rate of 59.7%, both of which are lower than that of the improved model. This means the model can more comprehensively capture target tomatoes in images and effectively reduce missed detections.

For the mean average precision at an intersection over union (IoU) of 0.5 (mAP@0.5), the improved model achieves 93.5%. It ranks first among all compared models. It not only significantly surpasses models like YOLOv8n but also outperforms models such as Faster R-CNN(65.7%), Cascade R-CNN(89.3%), SSD-MobileNetv2 (73.3%), SSD-MobileNetv3 (80.1%), EfficientDet-D0 (77.9%), and Nanodet (82.4%). This indicates more accurate target localization and recognition under the conventional IoU standard.

In terms of mAP@0.5:0.95, the improved model scores 58.6%. This score is higher than that of most compared models, including Faster R-CNN (26.3%) and Cascade R-CNN (53%), SSD-MobileNetv2 (39.3%), SSD-MobileNetv3 (46.4%), EfficientDet-D0 (43.1%), and Nanodet (46.5%). It demonstrates strong generalization ability and detection stability under different IoU standards. However, this metric of the improved model is slightly lower than that of YOLOv8s (61.6%), and the core reason lies in the differences in architecture and design objectives between the two models. YOLOv8s is supported by a parameter scale of 11.13 ×10^6^ (approximately twice that of the improved model’s 5.78 ×10^6^) and a computational complexity of 28.4G FLOPs (approximately 1.5 times that of the improved model’s 19.4G FLOPs). It captures fine-grained features of tomatoes through deeper C2f modules and wider feature channels, and without the constraint of a lightweight design, it can fully retain edge features to optimize the localization accuracy of medium-sized tomatoes, which account for 65% of the test set. In contrast, the improved model takes adaptation to embedded picking robots as its core goal. Its lightweight design, with parameters only 52% of those of YOLOv8s, limits feature expression. Although the newly added P2 layer optimizes the detection of extremely small targets, it introduces background noise during feature fusion. Additionally, the Slim-Neck structure also leads to the loss of some fine-grained features, and these two factors together affect the bounding box accuracy under high IoU thresholds. This performance gap represents a reasonable trade-off between “lightweight deployment” and “general high-precision performance”. The improved model is more in line with the low-cost deployment requirements of agricultural automation. It also outperforms most lightweight models in key tomato detection scenarios such as complex lighting and multi-fruit overlap. Moreover, the SPD convolution and PPA attention mechanism further enhance the ability to distinguish features between fruits and the background, reducing false detections and missed detections, which verifies the practical value of the proposed improvement scheme.

Regarding parameters and computational load, the improved model has 5.78 ×10^6^ parameters and a computational load of 19.4G FLOPS. Faster R-CNN has 137.10 ×10^6^ parameters and a computational load of 402.2G FLOPs. Cascade R-CNN has 65.4 ×10^6^ parameters and a computational load of 196G FLOPs. Although the improved model’s number of parameters is higher than that of lightweight models like SSD-MobileNetv2, SSD-MobileNetv3, Nanodet, YOLOv5n, and YOLOv8n, it is still much lower than that of Faster R-CNN and Cascade R-CNN. Compared with YOLOv8s (11.13 ×10^6^) and YOLOv8m (25.84 ×10^6^), it is also within a reasonable range. Its FLOPS are lower than those of models like YOLOv8s (28.4G) and YOLOv8m (78.7G), and far lower than those of Faster R-CNN and Cascade R-CNN. This shows that compared with models that have higher parameters or computational load but similar performance, the improved model has more advantages in terms of computational resource efficiency.

**Figure 13:**
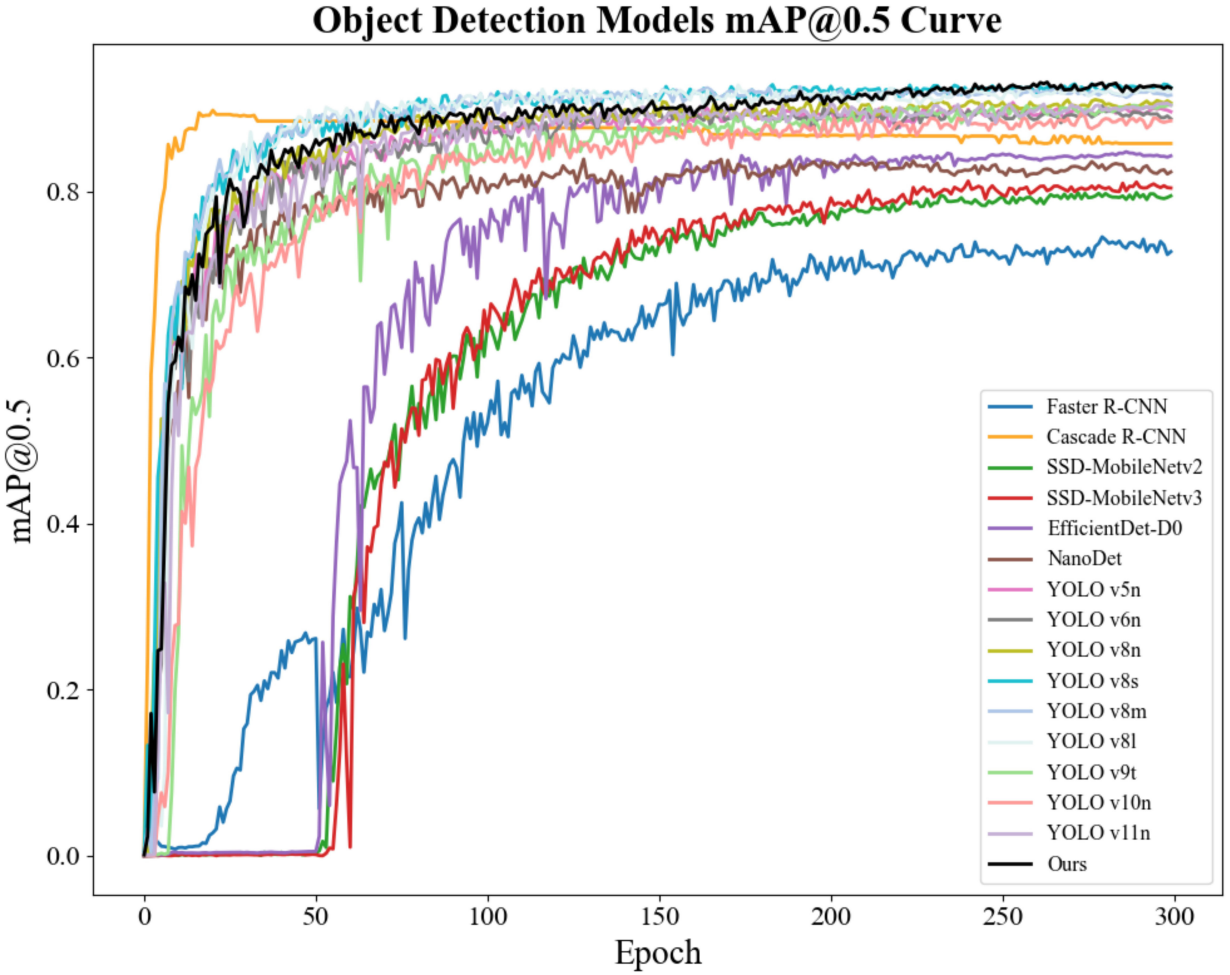
Mean average precision curves of various models.

In summary, the improved model performs excellently in detection accuracy. Its precision, recall rate, mAP@0.5, and mAP@0.5:0.95 all rank among the top of mainstream models. At the same time, compared with models like SSD-MobileNetv2, SSD-MobileNetv3, EfficientDet-D0, and Nanodet, it also has a significant improvement in comprehensive performance. Moreover, compared with two-stage algorithms like Faster R-CNN and Cascade R-CNN, it achieves better detection performance with much fewer parameters and lower computational load. Meanwhile, it balances the consumption of computational resources well, showing remarkable comprehensive advantages. This makes it more suitable for practical tomato detection tasks. To visually observe the performance of the improved model, **Figure 13** plots the mAP@0.5 curves of all models. Although its average precision is slightly lower than that of other models in the first 200 epochs, it surpasses all compared models between 200 and 300 epochs.

#### 5.3.3 Comparative experiment on model performance in complex weather

The generalization ability in open-field environments is a key factor restricting the practical application of tomato detection models. Unlike controlled greenhouse environments, open fields are often affected by uncontrollable natural weather conditions such as light, sudden rain, fog, and snow. These conditions easily distort image features and thus lead to the degradation of model detection accuracy. This bottleneck directly limits the model’s application in open-field precision agriculture. Breaking through this limitation will significantly expand the model’s application scope in precision agriculture and provide technical support for the in-depth integration of visual detection technology into the open-field agricultural production process.

To verify the robustness of the improved YOLOv8n model under complex weather conditions in open fields and its cross-domain adaptability from greenhouses to open fields, this study simulated five typical complex weather scenarios (Intense illumination, Overcast day, Rainy day, Snowy day, Foggy day) on the ‘Tomato’ dataset through data augmentation. Each weather scenario contains 895 images, and all are designed based on the actual interference characteristics of open-field environments:

##### 5.3.3.1 Simulation and parameter design of the intense illumination scenario

In the simulation of the Intense Illumination scenario, this study constructed a lighting environment covering typical high-light conditions of open fields in summer by setting a 3-level fixed brightness enhancement gradient and combining it with the adjustment of contrast and color parameters. Each scenario’s parameters are accurately matched to the actual agricultural environment, and the details are as follows:

Increase the image brightness by 40% while controlling the contrast within the range of 10%–20% to simulate a condition where the natural light intensity is 1.4 times that of the “standard agricultural environment”. The corresponding actual light intensity is 80,000–120,000 lux. Adjust the color parameters simultaneously: hue shift ±5, saturation increased by 5%–15%, and lightness increased by 10%–20% to reproduce the characteristics of fruit surface reflection and color perception deviation under intense light.

Increase the brightness by 50% and set the contrast to 15%–25% to simulate a condition where the light intensity is 1.5 times that of the standard environment. The actual light intensity ranges from 90,000 to 130,000 lux, with saturation increased by 8%–18% and lightness increased by 12%–22% to further approximate the real visual interference under moderately intense light.

Increase the brightness by 60% and adjust the contrast to 20%–30% to simulate a condition where the light intensity is 1.6 times that of the standard environment. The actual light intensity is 100,000–140,000 lux. Set the color parameters to hue shift ±7, saturation increased by 10%–20%, and lightness increased by 15%–25% to accurately reproduce the visual environment under high-intensity light.

The above three-level scenarios cover the common fluctuation range of intense light intensity in open fields.

##### 5.3.3.2 Simulation and parameter design of the overcast day scenario

In the simulation of the Overcast Day scenario, this study constructed a low-light environment that fits the typical overcast working conditions of open fields by fixing the brightness reduction parameters and combining them with the adjustment of contrast and color parameters. The scenario parameters are accurately matched to the actual agricultural environment, and the details are as follows:

Reduce the image brightness by a fixed 30% while controlling the contrast within the range of 10%–20% to simulate an overcast condition where the natural light intensity is 70% of the “standard agricultural environment”. The corresponding actual light intensity is 35,000–49,000 lux. Adjust the color parameters simultaneously: fine-tune the lightness by -15%–20% and fine-tune the saturation by -10%–10% to reproduce the characteristics of reduced color saturation and dim overall lightness in the overcast environment, and approximate the visual interference of real overcast scenarios.

##### 5.3.3.3 Simulation and parameter design of the rainy-day scenario

In the simulation of the Rainy Day scenario, this study constructed a simulated environment that fits the actual agricultural production conditions by distinguishing two typical rain intensities (moderate rain and heavy rain) and accurately matching the meteorological characteristics and visual interference parameters under each rain intensity. All scenario parameters are highly consistent with the actual characteristics of rainy days in this region. The specific settings are as follows:

In the simulation of the moderate rain scenario, the raindrop shape parameters are set to a diameter of 0.8–1.2 mm and a length of 2.7–3.3 mm before landing. In terms of lighting conditions, a brightness coefficient of 0.85 is used to simulate the cloud shading effect, maintaining the light intensity at 30,000–50,000 lux (approximately 50%–70% of that on sunny days). This conforms to the actual visual perception of “soft light and distinguishable crop details” in the moderate rain environment. A blur value of 3 px is used to simulate the slight light scattering effect under this humidity, further improving the authenticity of the scenario. In addition, based on field observation data, it is set that rain lines only cause a slight occlusion of 5%–15% on the edge of leaves and local parts of fruits, avoiding excessive interference with the key recognition features of crops.

In the simulation of the heavy rain scenario, the raindrop shape parameters are adjusted to a diameter of 1.5–2.5 mm and a length of 5.25–6 mm before landing to accurately reproduce the visual characteristics of heavy rainfall. In terms of lighting, a brightness coefficient of 0.75 is used to simulate the double shading effect of clouds and rain curtains, maintaining the light intensity at 15,000–30,000 lux (only 30%–60% of that on sunny days). This conforms to the actual characteristic of “visible crop outline but blurred details” in the heavy rain environment. A blur value of 5 px is used to reproduce the strong scattering effect, simulating the interference of rainwater on light propagation. In terms of occlusion effect, it is set that rain lines can cover 1/3–1/2 of the leaf width, forming the effect of “local feature occlusion but overall outline retention”. This not only reflects the interference of heavy rain on detection but also conforms to the visual presentation of crops in the actual planting scenario.

##### 5.3.3.4 Simulation and parameter design of the snowy day scenario

In the simulation of the Snowy Day scenario, this study constructed a simulated environment that fits the actual agricultural production conditions by distinguishing two typical meteorological snowfall levels (light snow and moderate snow) and accurately matching the meteorological characteristics and visual interference parameters under each snowfall level. All scenario parameters are highly consistent with the actual characteristics of winter snowy days in this region. The specific settings are as follows:

In the simulation of the light snow scenario, the horizontal visibility is set to be ≥ 1,000 meters. In terms of visual adjustment, the image brightness is slightly increased (slightly higher than that on sunny days but without dazzling reflection), and the color saturation is slightly reduced to present a soft tone. The image only has an extremely slight cool tone tendency (no excessive color cast). Within 5 meters nearby, the leaf texture of crops and the surface details of fruits are clearly distinguishable. Snowflakes adhere to the plant surface in sparse dots without forming a covering layer. Scenery beyond 50 meters only shows a slight “granular feeling” due to a small number of snowflakes in the air, which effectively avoids blocking the key recognition features of crops and conforms to the actual scenario of “normal operation of basic field work” under this snowfall level.

In the simulation of the moderate snow scenario, the horizontal visibility is set to be approximately 500–1,000 meters. In terms of visual adjustment, the image brightness is controlled within a reasonable range of “slightly lower than that on sunny days but higher than that in heavy snow”, the color saturation is significantly reduced, and the image shows a cool tone shift of -2 to -5 (in line with the visual characteristics of a low-temperature environment). Within 3 meters nearby, the main outline of the crops is clear with only thin snow covering the edges. The outline of plants 10–20 meters away becomes hazier, and the scenery beyond 30 meters only shows a rough outline. This not only objectively reflects the degree of interference of moderate snow on detection but also effectively retains the key recognition features of crops, conforming to the actual scenario of “close-range operation required for fine agricultural work” under this snowfall level.

In the simulation of the heavy snow scenario, the horizontal visibility is set to be ≤ 100 meters. In terms of visual adjustment, the image brightness is controlled within the range of “slightly lower than that on sunny days but not overly dark due to snow reflection”. The color saturation is significantly reduced to present a pale tone, and the image shows an obvious cool tone tendency (in line with the visual characteristics of a freezing low-temperature environment). Within 1–2 meters nearby, the general shape of crops is distinguishable, but their surfaces are covered with thick snow. The outline of plants 3–5 meters away is hazy, and the row spacing needs careful identification to distinguish. Scenery beyond 10 meters only shows blurred color blocks or rough outlines. This not only objectively reflects the strong interference of heavy snow on detection but also retains the core recognition features of crops, conforming to the actual scenario of “almost impossible to carry out fine agricultural work” under this snowfall level.

##### 5.3.3.5 Simulation and parameter design of the foggy day scenario

In the simulation of the Foggy Day scenario, this study constructed a simulated environment that fits the actual agricultural production conditions by distinguishing two typical fog concentration levels (light fog and dense fog) and accurately matching the meteorological characteristics and visual interference parameters under each fog condition. All scenario parameters are highly consistent with the actual characteristics of foggy days in this region. The specific settings are as follows:

In the simulation of the light fog scenario, the scenario visibility is set to 500–1,000 meters. In terms of visual adjustment, the image brightness is moderately reduced (slightly lower than that on sunny days but without obvious dimness), and the color saturation is slightly weakened to present a soft tone while fully retaining the basic color of crops. Within 10 meters nearby, the leaf texture of crops and the surface details of fruits are clearly distinguishable. Scenery beyond 50 meters only has slightly blurred edges, which avoids excessive interference with the key recognition features of crops and conforms to the actual scenario of “normal operation of basic field work” under these fog conditions.

In the simulation of the “dense fog” scenario, the scenario visibility is set to 200–500 meters. In terms of visual adjustment, the image brightness is significantly reduced (showing a soft dimness but without affecting the recognition of the main body of crops), and the color saturation is significantly weakened to present an elegant tone without changing the basic color of crops. Within 5 meters nearby, the main outline of the crops is clear. Scenery beyond 30 meters only shows a rough silhouette. The outline of plants 10–20 meters away is hazy, and their details are obviously weakened. This not only objectively reflects the degree of interference of dense fog on detection but also effectively retains the key recognition features of nearby crops, conforming to the actual scenario of “close-range operation required for fine agricultural work” under this fog condition.

Subsequently, we systematically evaluated the core indicators (Precision, Recall, mAP@0.5, mAP@0.5:0.95) of the improved YOLOv8n under each weather scenario. We also conducted a horizontal comparison with the Original Greenhouse Scenario (where the mAP@0.5 of the YOLOv8n model is 90.3%) to quantify the magnitude of performance degradation, and the comparison results are summarized in **Table 9**.

**Table 9:** Comparison of key metrics for the improved YOLOv8n model across different extreme weather scenarios in tomato detection tasks, with precision, recall, mAP@0.5, and mAP@0.5:0.95 as evaluation indicators.

Extreme weather scenarios	Model type	P%	R%	mAP@0.5/%	mAP@0.5:0.95/%
Original Greenhouse Scenario	YOLOv8n	85.5	84.5	90.3	56.6
Mild Intense Light	Ours	87.1	79.6	89.0	54.8
Moderate Intense Light	Ours	87.3	81.5	88.0	54.1
High-Intensity Light	Ours	86.5	79.8	87.0	52.4
Overcast Day	Ours	86.6	70.2	80.7	47.8
Moderate Rain	Ours	92.9	81.3	91.2	57.7
Heavy Rain	Ours	90.4	80.5	90.6	54.8
Light Snow	Ours	85.7	82.2	88.4	55.1
Moderate Snow	Ours	86.2	84.2	89.0	55.0
Heavy Snow	Ours	85.0	83.1	88.4	55.2
Light Fog	Ours	85.4	72.2	81.8	45.9
Dense Fog	Ours	82.2	70.0	78.0	45.1

With mAP@0.5 as the core, we quantified the performance degradation of each scenario relative to the greenhouse scenario. Meanwhile, combined with the changes in Precision (accuracy) and Recall (recall rate), we conducted an in-depth analysis of the model’s detection characteristics under different complex environments:

For the intense illumination scenario: From Mild Intense Light to High-Intensity Light, the Precision of the improved model is 87.1%, 87.3%, and 86.5% in sequence, maintaining a relatively high level overall. This indicates that the accuracy of the model in “positive sample determination” is less affected under intense illumination. The Recall values are 79.6%, 81.5%, and 79.8%, showing the characteristic that “the recall rate is slightly higher under Moderate Intense Light and slightly lower under Mild Intense Light and High-Intensity Light”. This reflects that the model has relatively better coverage capability for tomato targets under Moderate Intense Light. In terms of mAP@0.5, it is 89.0% under Mild Intense Light, with a degradation of 1.3% compared to the greenhouse scenario. It is 88.0% under Moderate Intense Light, with a degradation of 2.3%. And it is 87.0% under High-Intensity Light, with a degradation of 3.3%. The overall pattern shows that “the stronger the light intensity, the more significant the degradation, but the degradation amplitude is all< 5%”. This indicates that the model can still maintain good detection performance stability under the typical high-light working conditions of open fields in summer.

For the overcast scenario: Under Overcast Day, the Precision of the improved model is 86.6%, the Recall drops to 70.2%, and the mAP@0.5 is 80.7%, with a degradation of 9.6%. The combined effect of low illumination and reduced color saturation leads to an increase in the “missed detection” probability of the model for tomato targets, making it a scenario with a relatively large degradation amplitude besides intense illumination scenarios.

For the rainy scenario: Under Moderate Rain, the Precision reaches 92.9%, the Recall is 81.3%, and the mAP@0.5 reaches 91.2%, which is an increase of 0.9% compared to the greenhouse scenario. This is because the occlusion of tomatoes by rainwater is relatively small under Moderate Rain, and the light conditions are more compatible with those during model training. As a result, the model’s accuracy and coverage in identifying positive samples are both improved, demonstrating the model’s excellent adaptability under the Moderate Rain scenario. Under Heavy Rain, the Precision is 90.4%, the Recall is 80.5%, and the mAP@0.5 is 90.6%, with a degradation of -0.3%. Although the rain curtain occlusion and light scattering are more severe under Heavy Rain, the model can still balance Precision and Recall well through adaptive learning of parameters such as raindrop morphology and light scattering. This reflects the model’s good adaptability to the “rain intensity-occlusion-performance” correlation.

For the snowy scenario: Under Light Snow, the Precision of the improved model is 85.7%, the Recall is 82.2%, and the mAP@0.5 is 88.4%, with a degradation of 1.9%. At this time, snowflakes are sparse, causing little occlusion to the key recognition features of tomatoes, so the model can accurately capture the targets. Under Moderate Snow, the Precision is 86.2%, the Recall is 84.2%, and the mAP@0.5 is 89.0%, with a degradation of 1.3%. As the snowfall increases, the edges of tomatoes begin to be covered with thin snow, but the model can still identify the targets through the main contour and color features, leading to a slight increase in Recall. Under Heavy Snow, the Precision is 85.0%, the Recall is 83.1%, and the mAP@0.5 is 88.4%, with a degradation of 1.9%. Although the snow coverage increases and the visibility decreases, the model still retains the ability to recognize the overall shape and basic color of tomatoes. The degradation amplitude gradually increases with the “increase in snow coverage and decrease in visibility”, but all are< 3%. This fully indicates that the model has strong robustness against snow interference.

For the foggy scenario: Under Light Fog, the Precision of the improved model is 85.4%, the Recall is 72.2%, and the mAP@0.5 is 81.8%, with a degradation of 8.5%. The slight scattering of fog blurs the details of distant tomatoes, resulting in a decrease in Recall, but the nearby targets can still be accurately identified. Under Dense Fog, the Precision is 82.2%, the Recall is 70.0%, and the mAP@0.5 is 78.0%, with a degradation of 12.3% (calculated by subtracting 78.0 from 90.3). Due to the “blurring-transmission” characteristic of fog, the propagation of light is seriously hindered, and a large number of key features of tomato targets (such as texture and color) are lost. As a result, both the Precision and Recall of the model decrease significantly. The degradation amplitude shows a strong negative correlation with visibility, and the degradation exceeds 10% under Dense Fog, making it the most challenging scenario for the model.

Further analysis of the applicable environmental range of the model reveals that when the environment meets the following conditions: “the light intensity corresponds to Mild to High-Intensity Light (actual light intensity 80,000–140,000 lux), where the light intensity is strong but does not cause excessive distortion of tomato features; Moderate to Heavy Rain (corresponding to light intensity 15,000–50,000 lux), where the rainwater occlusion and light scattering are within the adaptive range of the model; Light to Heavy Snow (horizontal visibility ≥ 100 meters), where the occlusion of key tomato features by snow is not severe; Light Fog (visibility 500–1,000 meters), where the damage to target details caused by fog scattering is limited”, the mAP@0.5 degradation of the improved YOLOv8n model is< 5%, and the Precision and Recall can also be maintained at a high level, enabling stable and accurate detection of tomatoes. Only under extreme environments such as “Overcast Day (light intensity 35,000–49,000 lux), where the combination of low illumination and dim color significantly reduces the recognizability of tomato features; Dense Fog (visibility 200–500 meters), where strong light scattering and target blurring make feature extraction difficult”, the performance degradation is relatively obvious. In such cases, it is necessary to apply the model carefully according to scenario requirements or match it with auxiliary sensing equipment during actual deployment to enhance detection capability.

## 6 Discussion

### 6.1 Analysis of detection performance in polymorphic scenarios

**Figure 14:**
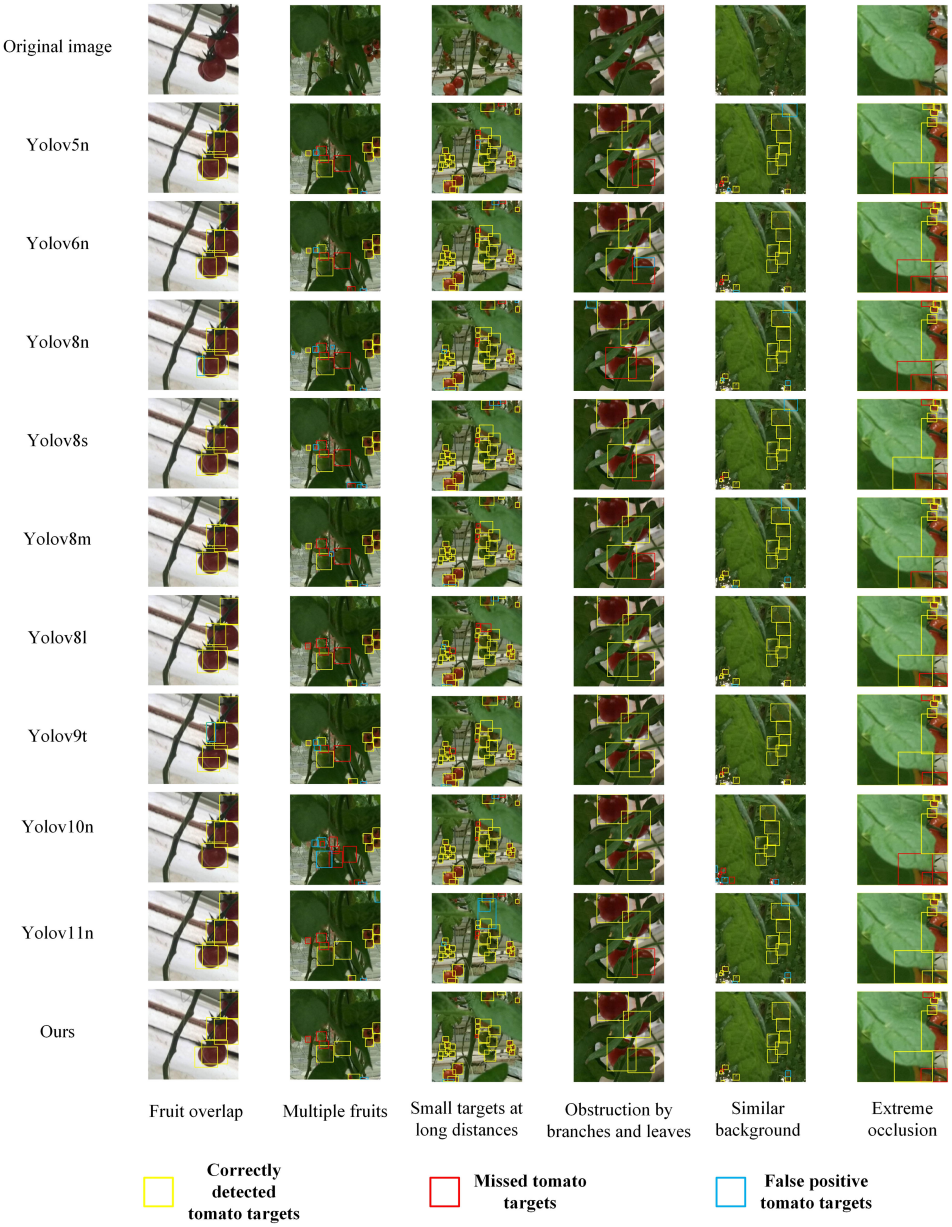
Comparison of tomato detection effects of various models in polymorphic scenarios.

Images from the test set that depict polymorphic and complex greenhouse environments were selected. They were used to compare the detection performance of different models across various scenarios. These scenarios include fruit overlapping, multiple fruits, distant small targets, branch occlusion, leaf occlusion, and extreme occlusion. **Figure 14** presents a comparison between the improved YOLOv8n model and other YOLO-series models. The other models are YOLOv5n, YOLOv6n, YOLOv8n, YOLOv8s, YOLOv8m, YOLOv8l, YOLOv9t, YOLOv10n and YOLOv11n.

YOLOv5n performed poorly in several scenarios. It could not accurately outline the contour of a single fruit in the case of fruit overlapping. It had target omissions in multi-fruit environments. Its ability to detect distant small targets was weak, leading to frequent omissions. It also missed some tomatoes occluded by branches or leaves and occasionally produced false detections. In extreme occlusion scenarios, the omission problem of YOLOv5n was extremely prominent. A large number of tomatoes deeply occluded by leaves were not detected. It could only identify tomatoes with little occlusion.

YOLOv6n had low accuracy in fruit overlapping scenarios, multi-fruit scenarios, and extreme occlusion scenarios. Its detection boxes were blurred. It had insufficient precision for distant small targets, which resulted in frequent omissions. It faced difficulties in detecting occluded tomatoes and was accompanied by a large number of false detection annotations.

YOLOv8n had a limited ability to distinguish overlapping regions. It had a large number of omissions in multi-fruit detection and extreme occlusion scenarios. It frequently missed distant small targets. Due to insufficient feature extraction, false detection was prominent when there was occlusion.

Although YOLOv8s, YOLOv8m, and YOLOv8l showed improved capabilities as the model size increased, they still struggled to distinguish fruit boundaries in fruit-overlapping scenarios. The completeness of their multi-fruit detection needed optimization. They had high omission rates for distant small targets. In addition, there were gaps in their precision and recall when there was occlusion, and the false detection situation varied among different models. For extreme occlusion, they performed better than smaller models, but their detection accuracy for targets with very little exposure was still not high, and there were still certain omission situations.

YOLOv9t, YOLOv10n, and YOLOv11n showed improvements in various scenarios. However, the accuracy and completeness of their fruit overlapping and multi-fruit detection still need to be enhanced. They could not solve the omission problem of distant small targets. In extreme occlusion situations, YOLOv9t and YOLOv10n still had omissions for highly occluded tomatoes. YOLOv11n could detect some occluded tomatoes that smaller models missed, and its robustness was improved to a certain extent.

Compared with the baseline model, the improved YOLOv8n significantly reduces distant small-target omission rate via SPD convolution and small-target detection layer synergy, while maintaining high small-tomato detection precision. The PPA attention and Detect_CBAM enhance anti-occlusion ability—better detecting branch/leaf-occluded tomatoes and having lower false detection rates in leaf occlusion. Visual results show more correct detection boxes and fewer omissions/false detections in most complex scenarios, highlighting good completeness and accuracy.

However, in extreme occlusion, detection difficulty rises. The model can locate tomatoes with larger exposed areas, but fails to detect an extremely small upper-right tomato and has positioning deviation for a severely occluded lower tomato.

Mechanistically, this limitation stems from the PPA local branch patch size being larger than “extremely small exposed area” pixels and SPD’s limited ultra-small target detail retention. Practically, the model identifies most core targets but misses those with extremely small exposed areas—this shows current performance boundaries and points to subsequent optimization: targeted module parameter adjustment or auxiliary technologies to expand ultra-extreme occlusion detection ability.

### 6.2 Verification of model robustness and cross-domain adaptability in complex weather scenarios

**Figure 15:**
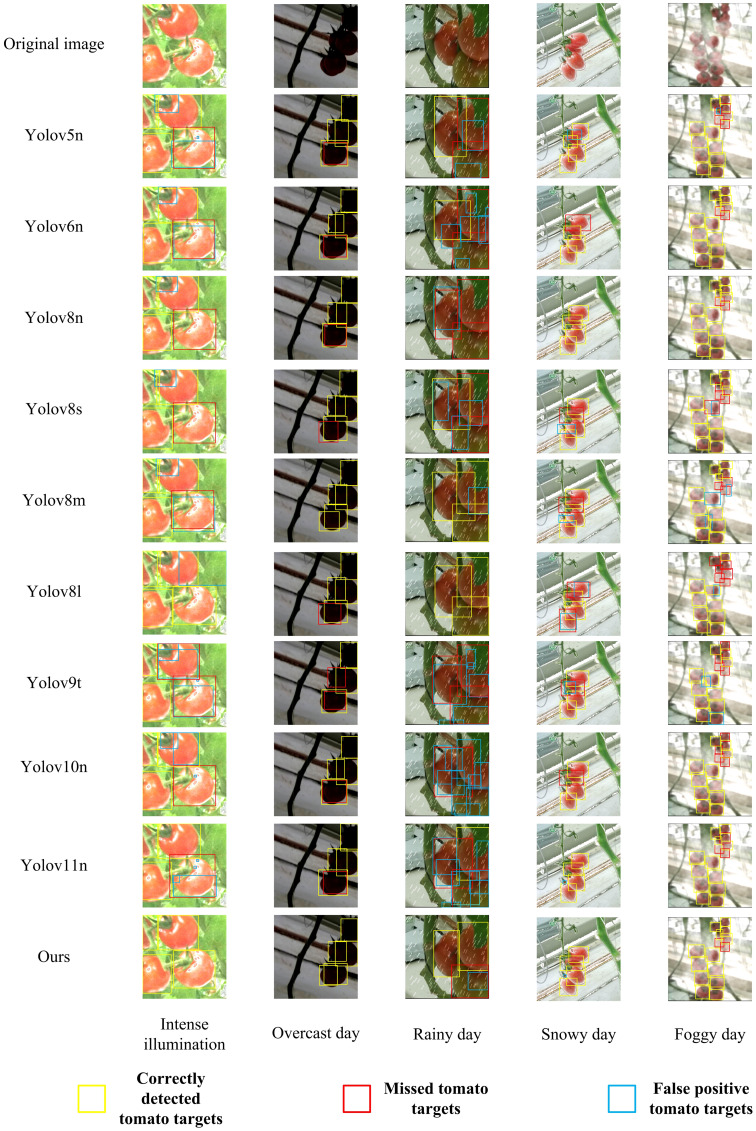
Comparison of tomato detection effects of various models in complex weather scenarios.

To intuitively present the detection performance of the improved YOLOv8n under complex weather conditions, this study specifically simulated typical complex climate scenarios common in open-field environments (including intense illumination, overcast days, rain, snow, and fog) using data augmentation technology. It clearly selected mild, intense light, moderate rain, moderate snow, and light fog as the core comparison scenarios. On this basis, aiming at these uncontrolled complex scenarios, visual analysis and comparison were conducted on the detection effects between the improved YOLOv8n and other mainstream YOLO series models (YOLOv5n, YOLOv6n, YOLOv8 n/s/m/l, YOLOv9t, YOLOv10n, YOLOv11n). The specific comparison results are shown in **Figure 15**.

In intense illumination, YOLOv5n had severe specular reflection covering fruit edges with highlights and causing missed detections. YOLOv6n failed to suppress light spots well, mistaking them for tomatoes and leading to heavy false detections. YOLOv8 series were affected by high-brightness regions, making the target-background contrast drop sharply and causing severe tomato missed detections. YOLOv9t, YOLOv10n, and YOLOv11n slightly improved high-brightness suppression but still had missed and false detections. The improved model used SPD convolution’s multi-scale filtering to better remove specular reflection interference with no missed detections.

In overcast conditions, low brightness and compressed contrast weakened fruit-leaf grayscale differences. YOLOv5n’s limited feature extraction reduced discrimination between similar-colored fruits and leaves, leading to blurred detection boxes and higher missed rates. YOLOv6n had no false detections but suffered from missed detections. YOLOv8 series, limited by poor target feature discriminability, mainly had missed detections without new false ones. Models like YOLOv9t had a weaker ability to detect tomatoes in grayscale backgrounds, with much higher missed rates than in other scenarios. The improved model relied on SPD convolution’s low-light detail capture and combined it with PPA attention’s multi-scale feature fusion to reduce low-contrast caused missed detections.

In rainy scenarios, rain streak and water droplet texture noise heavily interfered with detection. YOLOv5n could not filter rain streaks well, leading to frequent missed detections when fruits were covered by raindrops. YOLOv6n had much higher false detection rates in rain curtains. The YOLOv8 series had obvious detection box drift due to rain streak noise. YOLOv9t and other models were sensitive to dense rain streaks, causing both missed and false detections. The improved model used PPA attention to suppress rain streak noise and Detect_CBAM’s spatial attention for localization to greatly reduce rain streak-induced detection deviations.

In snowy conditions, snowflakes on white backgrounds created strong interference. YOLOv6n had false detections due to extremely low target contrast under white reflections. YOLOv8 series failed to extract low-contrast features well in snow, leading to tomato missed detections or incomplete boxes. YOLOv9t, YOLOv10n, YOLOv11n, and other models had much lower recall rates under white background interference. The improved model used SPD convolution’s edge enhancement to strengthen tomato contour features in snow and combined channel attention to focus on color features, improving target discriminability.

In foggy scenarios, fog made images hazy and edges weak. YOLOv5n had high target missed rates due to low visibility. YOLOv6n had no false detections but obvious missed detections. YOLOv8 series often mistook fog clusters for fruits, causing frequent false detections. YOLOv9t, YOLOv10n, YOLOv11n, and other models had poor adaptability to foggy low contrast, leading to lower overall detection accuracy. The improved model used Detect_CBAM’s channel-spatial dual attention to focus accurately on fruit regions, avoiding fog cluster misjudgment.

Visualization showed that the improved YOLOv8n had far more yellow correct detection boxes than other models in all five weather scenarios. It also had many fewer red missed detection boxes and blue false detection boxes. This fully confirms its robustness and cross-domain adaptability in complex weather, laying a solid foundation for its practical use in uncontrolled agricultural environments like open fields.

### 6.3 Analysis of feature attention mechanisms and generalization capability

In the process of agricultural automation and intelligence, the detection of tomatoes throughout their growth cycle in greenhouse environments is a core component for achieving precise production management. While optimizing the detection performance for mature tomatoes, this study expands the detection capability of the improved YOLOv8n model to include immature fruits through architectural modifications, a feature of critical significance for yield prediction, nutritional regulation, and harvesting schedule planning. To analyze the model’s decision-making logic in polymorphic scenarios, Grad-CAM (Selvaraju et al., 2016; Chattopadhyay et al., 2017) technology was employed to generate target detection heatmaps, visualizing the model’s attention distribution across image regions and revealing its feature capture mechanisms and generalization capabilities.

Grad-CAM calculates gradients of target-category feature maps from the final convolutional layer, processes them via global average pooling and ReLU activation, and maps the model’s decision basis to pixel-level heat response maps. This method does not require modifications to the network structure or retraining, applies to various CNN architectures, and can intuitively locate key regions of model attention—the deeper red areas contribute more to detection, followed by yellow areas, while blue areas are regarded as background redundant information. This visualization tool provides an interpretability basis for model debugging and performance optimization.

**Figure 16:**
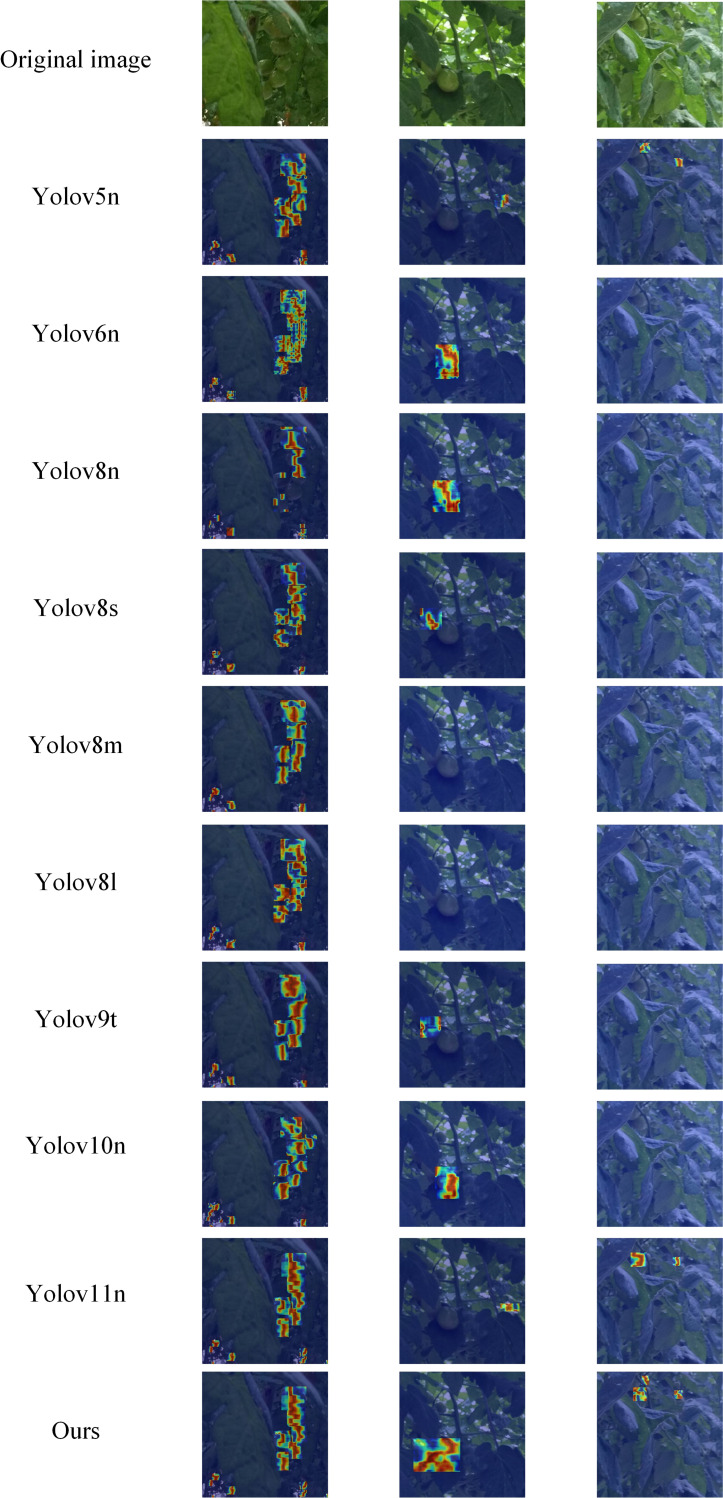
Visualization of heatmaps for various models.

As shown in **Figure 16**, traditional YOLO models have clear heatmap flaws. YOLOv5n has discrete heat responses with blurred target-background boundaries, struggling to suppress interference. YOLOv6n covers fruit bodies but focuses heat on local high-contrast areas, relying on single cues with incomplete extraction for irregular fruits. YOLOv8n shows weak heat responses and offset hotspots in small targets, causing poor feature capture and positioning. Larger YOLOv8s/m/l face issues with feature efficiency decoupling and fine retention. Even newer YOLOv9t/10n/11n fail to solve core greenhouse problems, over-reliance on single features, unstable positioning, and poor multi-scale adaptability. These flaws highlight key optimization directions for our improved model.

This study reveals the improved model’s anti-interference mechanisms and multi-stage detection potential through heatmap visualization, providing a typical case for interpretability analysis of deep learning models in agricultural scenarios. Its performance in full-growth-cycle detection lays a technical foundation for the intelligent management of greenhouse tomatoes.

### 6.4 Analysis of picking application value

the improved model in this study demonstrates three core values in practical tomato picking scenarios, particularly providing key technical support for automated picking, and is significantly superior to existing detection methods:

1. Traditional models generally have low recall rates for small targets and occluded fruits in polymorphic environments. Through the collaborative optimization of the added small-object detection layer and SPD convolution module, this study has increased the recall rate to 87.3%, with the most significant improvement in the detection rate of small fruits. Notably, the PPA attention mechanism plays a key role here: its local branch can capture the residual local features of tomatoes, such as calyx texture and fruit edge gradients, under extreme occlusion, while the global branch screens the spatial correlation between dense tomato clusters and the background—this dual-branch design ensures that even tomatoes with only a tiny portion of their fruit surface exposed are not missed. When applied to robotic arm picking in the future, it can reduce the missed picking of mature fruits and enhance picking completeness.
2. In automated picking, if the model has false detections. The self-developed Detect_CBAM detection head in this study has significantly reduced the false detection rate through channel and spatial attention mechanisms, especially in leaf-dense areas, where the ability to distinguish “fruit-leaf” features has been significantly improved. Specifically, the channel attention of Detect_CBAM calculates the weight of tomato color channels, and this weight is much higher than that of leaf channels, effectively suppressing leaf feature interference; the spatial attention further locates the tomato position by analyzing the pixel contrast of local areas, avoiding the misjudgment of leaf shadows as tomatoes. When combined with robotic arms in the future, it can reduce invalid actions and fruit damage, and improve picking quality.
3. Existing high-precision models (e.g., YOLOv8m) feature large parameter scales and high computational costs, making them difficult to adapt to the hardware resources of low-cost picking robots. This study conducts a lightweight design on the feature extraction layer of the model. On the premise of ensuring detection accuracy, it reduces hardware requirements and has good adaptability to scenarios such as complex lighting conditions and dense fruits in greenhouses, thus providing technical feasibility for the large-scale application of robotic arm picking equipment in the future.

### 6.5 Future research directions

This section discusses the improved YOLOv8n’s performance in tomato detection under complex greenhouse environments. Via comparative analysis and visualization, we verified the improvement strategies’ positive effects— the model showed higher accuracy, completeness, and robustness in polymorphic scenarios. Heatmap analysis also revealed its precise attention to target regions, confirming generalization in polymorphic agricultural scenarios. Despite significant performance gains, the model has boundaries: in extreme occlusion, it still misses tomatoes with only pixel-level tiny exposed surfaces or deep occlusion. This objectively presents current limitations and clarifies subsequent optimization directions. Several potential improvement directions still need further exploration:

#### 6.5.1 Deepening full-growth-cycle detection

This study verified the model’s potential to detect immature fruits via Grad-CAM, but color confusion between immature fruits and leaves persists. Future research can expand datasets and explore multi-task learning frameworks—integrate time-series growth data with phenotypic parameter association analysis to enhance feature discrimination of fruits at different growth stages, improve full-cycle detection accuracy, and provide quantitative indices for high-throughput breeding screens. This capability can also be extended to broader phenotyping applications like dynamic monitoring of daily fruit growth rates across crops.

#### 6.5.2 Expand small-target detection techniques and optimize the model’s adaptability to extreme occlusion scenarios

While the current model improves distant small-target detection via SPD convolution and small-target detection layer collaboration, it faces two challenges: extremely small-pixel targets lack sufficient features; in extreme occlusion, tomatoes deeply covered by leaves expose minimal pixel surfaces, and existing mechanisms fail to activate their responses. Future work can introduce super-resolution preprocessing to enhance tiny region details, reduce PPA attention’s local branch patch size for tiny feature capture, and adjust Detect_CBAM’s spatial attention weights. Targeted module parameter optimization or auxiliary technologies can break extremely small-target detection bottlenecks and expand the model’s capability in ultra-extreme occlusion.

#### 6.5.3 Verification of lightweight deployment versatility and edge device adaptability

This study lightened the model via Slim-Neck, but embedded edge deployment is incomplete, hardware driver adaptation, and real-time inference: optimization still needs breakthroughs.: Future work will adopt knowledge distillation, using the current lightweight model as a teacher network to train a more streamlined student network and reduce edge device load, optimize model operators based on edge hardware computing architecture to improve inference compatibility, and conduct comparative experiments on different edge devices to test practical inference metrics, clarify optimal deployment schemes for different hardware, and advance algorithm-hardware collaborative adaptation research in resource-constrained scenarios.

#### 6.5.4 Investigating collaborative mechanisms between algorithms and agricultural equipment

The current model focuses on visual detection and has not been integrated with agricultural production equipment. Extending the improved algorithm to tomato-picking robots in future research—exploring applications of detection results in practical operations such as robot path planning and fruit grasping—will facilitate the transformation of research outcomes into agricultural automation scenarios and boost agricultural production efficiency.

#### 6.5.5 Fusion of multimodal agronomic parameters

Future research can integrate hyperspectral imaging and thermal infrared data into the existing detection framework, combine it with the PPA attention mechanism’s multi-scale feature analysis to build models for fruit external and intrinsic quality prediction. By analyzing the coupling law between fruit surface temperature distribution and color changes, it can accurately predict harvest time and quality grading. Meanwhile, an open phenotyping dataset covering fruit developmental sequences of multiple varieties and environments can be established to support algorithm generalization verification. This provides key technical support for precision agriculture growth regulation and breeding, and aligns with broader phenotyping applications like crop monitoring, plant health assessment, and breeding-related trait extraction.

#### 6.5.6 Explore the generalized application of the improved strategies in multi-crop detection

The proposed PPA attention and self-designed Detect_CBAM detection head work well in tomato detection, and their ability to address occlusion and small-target issues can be extended to strawberries, grapes, etc., with three specific promotions. First, adaptive optimization for target crops: for small-fruit crops, adjust PPA local branch patch size from p=2 to p=1 to match tiny fruit stalk features and optimize Detect_CBAM channel attention to strengthen color differences between strawberry surfaces and leaves; for clustered crops, optimize PPA global branch spatial correlation calculation and adjust Detect_CBAM spatial attention receptive field to avoid dense berry detection box overlap. Second, build a multi-crop shared dataset: collect data of strawberry leaf occlusion, grape cluster overlap, etc., label unified indicators, and record Detect_CBAM’s cross-crop key parameters to provide data and parameter support. Third, lightweight generalization verification: integrate Slim-Neck, PPA, and Detect_CBAM into other crop models, verify edge device performance, and confirm contribution via ablation experiments to improve strategy universality.

## 7 Conclusion

To address the challenges of tomato detection in polymorphic greenhouse environments, this study presents an enhanced tomato detection method based on an improved YOLOv8n architecture. Through collaborative optimization of multiple modules and data augmentation strategies, the proposed method significantly improves detection accuracy and model generalization, providing reliable technical support for automated tomato picking. The main conclusions are as follows:

1. This study used image data augmentation to boost the greenhouse tomato dataset diversity and model generalization. It introduced the SPD convolution module for lossless downsampling between Conv and C2f modules and added non-strided convolutions to enhance small-target feature extraction. A dedicated small-target detection layer fused with the PPA attention mechanism, using 160×160-pixel large-scale feature maps and multi-branch capture, reduced missed detections in occluded or overlapping scenarios to ensure automated picking completeness. The VoV-GSCSP structure replaced the traditional neck network, simplifying layers and parameters to cut model complexity and computational demands. The Detect_CBAM head used channel-spatial attention to enhance tomato features, suppress background interference, and reduce invalid robotic picking. The DIoU loss function optimized bounding box regression, accelerated convergence, and provided more accurate coordinates for picking path planning.
2. Comparative experiments demonstrate that after training on the tomato_dataset, the improved model significantly outperforms the YOLOv8n baseline model and mainstream comparative models in terms of comprehensive performance. Its precision, recall, mAP@50, and mAP@50:95 is respectively improved by 2%, 2.5%, 3.0%, and 1.3% compared to YOLOv8n. Visualization analysis further highlights its advantages: in polymorphic scenarios such as fruit overlapping and distant small targets, the improved model exhibits higher accuracy in detection bounding boxes, more complete target recognition, effectively reduced missed detections, and precise localization of small targets. Grad-CAM heatmaps reveal that the model can suppress background interference and focus on tomato target regions in polymorphic scenarios.

This study provides a visual detection solution for intelligent tomato picking that balances accuracy and efficiency. Through multi-module collaborative optimization and data augmentation strategies, it breaks through the technical bottlenecks of tomato detection in polymorphic greenhouse environments, offers a reusable technical paradigm for the research and development of agricultural intelligent equipment, and also provides an important reference for the automated detection and picking of other fruits and vegetables, promoting the practical application process of automated agricultural detection technologies.

## Data Availability

The original contributions presented in the study are included in the article/Supplementary Material. Further inquiries can be directed to the corresponding author/s.
